# Controlling the pH-response of branched copolymer nanoprecipitates synthesised by transfer-dominated branching radical telomerisation (TBRT) through telogen chemistry and spatial distribution of tertiary amine functionality†

**DOI:** 10.1039/d2na00399f

**Published:** 2022-08-22

**Authors:** Oliver B. Penrhyn-Lowe, Savannah R. Cassin, Pierre Chambon, Steve P. Rannard

**Affiliations:** Department of Chemistry, University of Liverpool Crown Street L69 7ZD UK srannard@liv.ac.uk; Materials Innovation Factory, University of Liverpool Crown Street L69 7ZD UK

## Abstract

Amine functionality offers the modification of polymer properties to enable stimuli-responsive behaviour, and this feature has been utilised in numerous studies of self-assembly and disassembly. The ability to place amines as pendant groups along linear polymer backbones within distinct blocks, at chain ends or as statistical mixtures with other functionalities, has allowed fine tuning of responses to pH. Here we study and compare the placement of amines within the backbones or as pendant groups within polyesters synthesised by the newly reported transfer-dominated branching radical telomerisation (TBRT). Branched polymers with backbone amines are clearly shown to undergo dissolution that is determined by pH and telogen selection; they undergo nanoprecipitation only when hydrophilic telogens are present within their structure and provide nanoprecipitates that are highly sensitive to the addition of acid. In contrast, TBRT polymers with pendant amines form uniform nanoparticles with remarkable stability to pH changes, under identical nanoprecipitation conditions. The behaviour differences shown here open new avenues of synthetic flexibility for pH-responsive polymer design using TBRT.

## Abstract 2

The pH response of macromolecules and nanoprecipitated particles formed from novel amine-containing branched polymers is studied with significantly varied behaviour seen from materials with different spatial distributions of amine groups.

## Graphical abstract

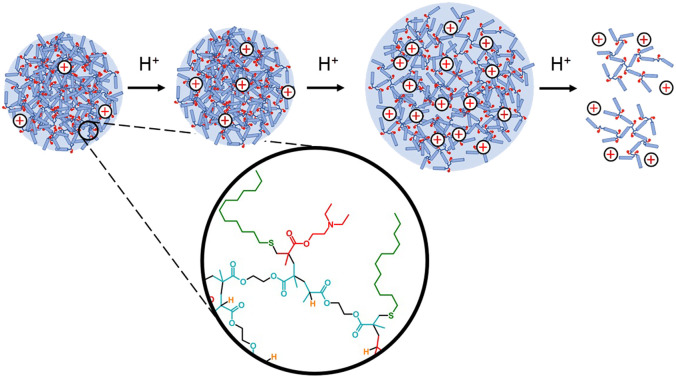

## Introduction

Stimuli responsive polymers have intrigued polymer chemists for many years and have underpinned a number of technologies within modern products. The range of stimuli that have been studied in the published literature includes magnetic fields,^1^ temperature,^2^ solvents,^3^ light,^4^ and pH,^5^ whilst the response of the polymers in question may be a triggered degradation,^6^ change in shape,^7^ or an assembly/disassembly^8^ process to name a small number of opportunities.

Within pH-responsive polymers, various functional groups allow tuning of the response towards different environmental conditions. For example, the pH-dependent polyelectrolyte behaviour of amine and acid containing polymers may lead to solvation in aqueous environments or a switch from the hydrophilic to hydrophobic character with a subsequent assembly or precipitation. Recent studies have shown, for example, that the reversible protonation/deprotonation of pH-responsive gels may dramatically alter interactions with proteins,^9^ whereas drug delivery to tumour sites may take advantage of the localised decrease in pH within the disease site.^10^

Amine functional polymers have been considerably studied as they are ideal candidates for pH-responsive polymer research. In most cases, polyamines formed by chain-growth mechanisms contain C–C backbones with pendant functionality and are generally derived from various polymerisations of (meth)acrylate, (meth)acrylamide, vinyl benzylamines, vinyl pyridines or vinyl imidazole monomers.^11^ Tertiary amine methacrylate polymers, and their quaternised derivatives, have been studied^12^ in various architectures such as linear homopolymers,^13,14^ block copolymers,^15–20^ crosslinked star copolymers,^21^ lightly branched copolymers,^22^ hyperbranched polydendrons,^23,24^ and shell crosslinked micelles.^25^

Recently, we have introduced the synthesis of high molecular weight branched polymers and copolymers using free radical techniques that had been previously directed towards the creation of telomers, namely transfer-dominated branching radical telomerisation (TBRT).^26^ Telomers were first described over 70 years ago^27,28^ and are very short chain polymeric structures containing between 2 and 5 repeat units and are formed by the addition of a telogen to a taxogen. In many cases, the telogen, A–B, acts as a chain transfer agent to limit the radical mediated reaction on unsaturated taxogens, Z, to form structures that incorporate all of the chemical components of the telogen and taxogen within the final telomer, A–[Z]_*n*_–B, where *n* is maintained at a very low value.^29,30^ Such approaches have been recently utilised to combine unsaturated natural product taxogens, such as terpenes, into a range of useful chemical building blocks using telogens such as alcohols, amines, thiols, carbon dioxide and carbon monoxide.^31–34^

**Fig. 1:**
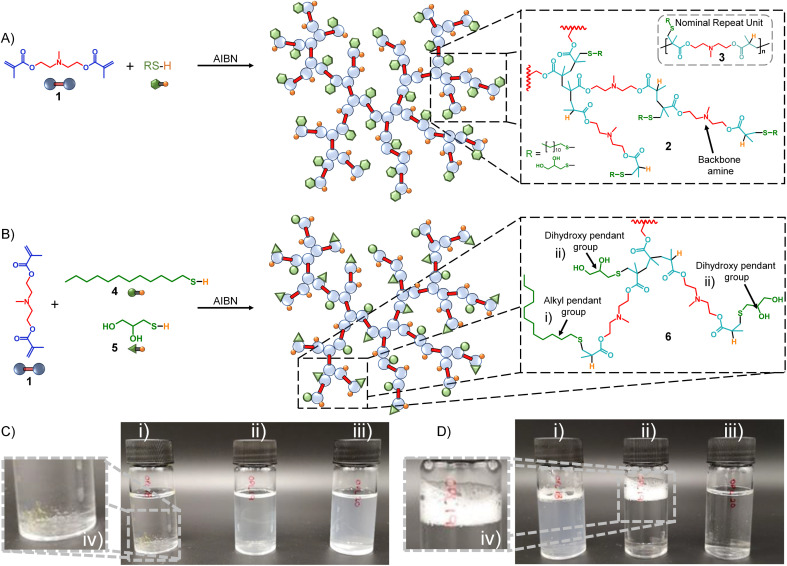
Schematic representations of TBRT polymers and aqueous solution behaviour. (A) General schematic of the TBRT synthesis of branched polyesters containing tertiary amines residing in the backbone of the complex architecture and using a single telogen; (B) general schematic of the synthesis of mixed telogen statistical copolymers with backbone amine functionality by TBRT; (C) photographs of the (i) p(DDT–BMEMA) homopolymer, (ii) p([TG–BMEMA]_0.5_-*stat*-[DDT–BMEMA]_0.5_) mixed telogen statistical copolymer, and (iii) p(TG–BMEMA) homopolymer added to water – (iv) shows the undissolved p(DDT–BMEMA) homopolymer. (D) Photographs of the (i) p(DDT–BMEMA) homopolymer, (ii) p([TG–BMEMA]_0.5_-*stat*-[DDT–BMEMA]_0.5_) mixed telogen statistical copolymer, and (iii) p(TG–BMEMA) homopolymer added to water after addition of HCl – (iv) shows stable foam formation from the p([TG–BMEMA]_0.5_-*stat*-[DDT–BMEMA]_0.5_) mixed telogen statistical copolymer (foaming behaviour was observed for all mixed telogen compositions). Expansions show architectural variation derived from statistical incorporation of telogens within the branched polymers.

**Fig. 2:**
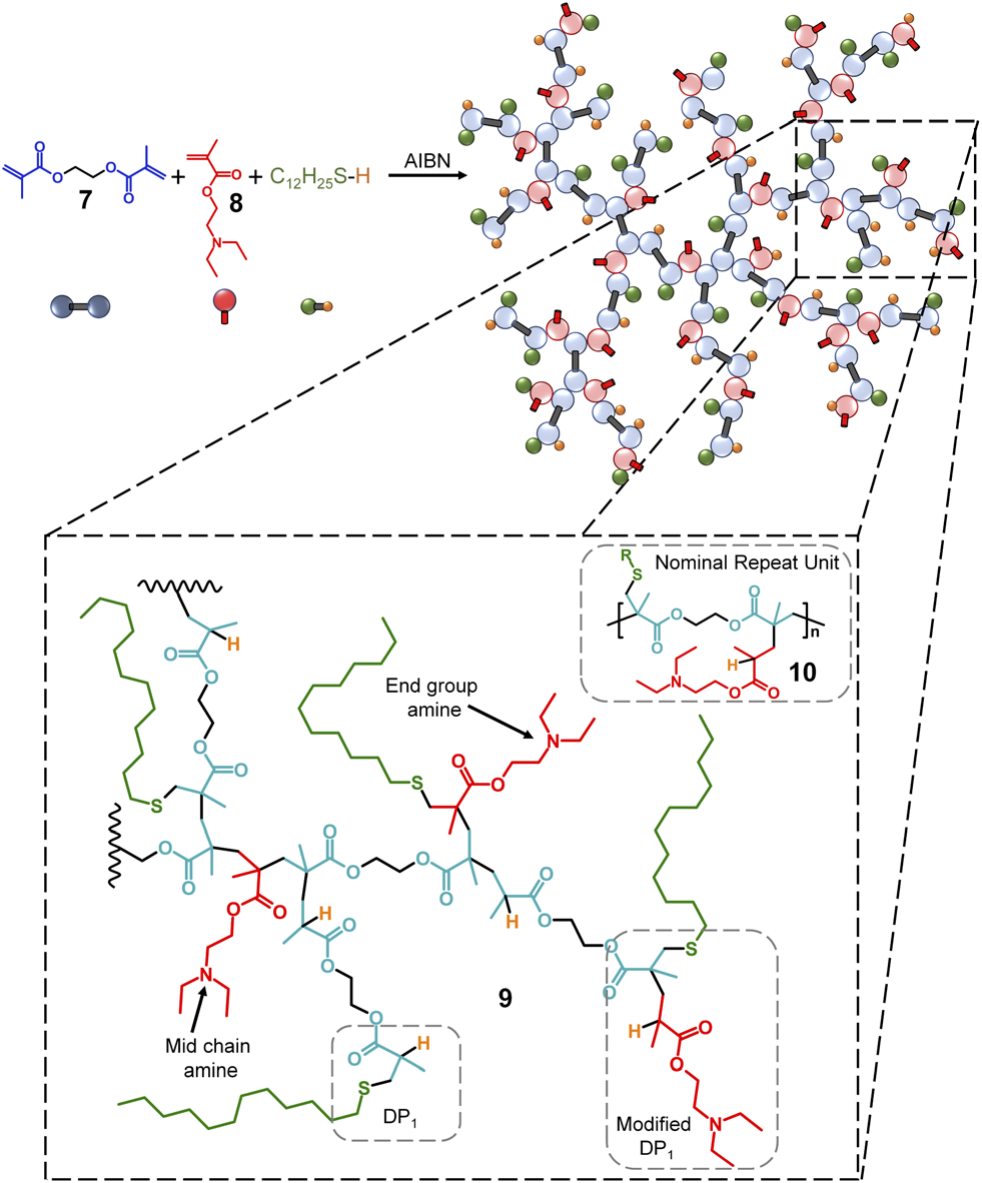
Schematic of the TBRT synthesis of a mixed mono-vinyl taxogen/MVT statistical copolymer using ethylene glycol dimethacrylate, 6, and 2-(diethylamino)ethyl methacrylate, 7. Expansion shows architectural complexity derived from the statistical incorporation of the mono-vinyl taxogen within the branched polymer.

In summary, TBRT uses similar free radical conditions that have been reported many times for the synthesis of linear telomers from mono-vinyl taxogens;^35–42^ however, the use of multi-vinyl taxogens (MVTs) in the presence of sufficient amounts of telogen to maintain a number average telomer length of <2 vinyl units, allows TBRT to generate very high molecular weight polymers from the homopolymerisation of MVTs, at full conversion of vinyl functional groups, without the formation of crosslinked gels.^25,43–45^ We have recently demonstrated new strategies to form novel statistical copolymers through TBRT chemistry. Mixed telogen feedstocks lead to the formation of polymers with statistically incorporated telogen-derived pendant groups, and we have called these mixed telogen statistical copolymers.^45^ Additionally, the introduction of low concentrations of mono-vinyl taxogens allows the statistical introduction of non-telogen-derived pendant groups, and we named these mixed mono-vinyl taxogen/MVT statistical copolymers.^45^ Importantly, TBRT utilises chain-growth chemistries to create branched polymers with backbones that are analogous to step-growth polymers through the creation of extended chains that comprise the chemistry of the MVT, rather than C–C chains formed through conventional long-range propagation of vinyl groups. For example, a dimethacrylate will form a branched polyester backbone. Here, we utilise this unusual ability of TBRT to form step-growth backbone polymers to synthesise tertiary amine containing branched polymers (Fig. 1A) with the amine functionality either within the polyester backbone (Fig. 1) or pendant to the polyester architecture (Fig. 2). This allows the importance of the placement of functional groups in the pH-responsive self-assembly and disassembly of nanoprecipitated polymer particles to be studied.

The synthesis of the dimethacrylate monomer *N*,*N*-bis(methacryloxyethyl) methylamine (BMEMA, 1) has been reported previously where it was employed to form core crosslinked cationic star polymers,^46^ and this synthesis was readily repeated (see ESI Fig. S1–S4†). Under TBRT conditions, BMEMA is used as an MVT and is homopolymerised to form an extended polyester-amine backbone, 2, within the branched architecture (Fig. 1A). The nominal repeating structure, 3, therefore, contains a backbone tertiary amine with pendant thioether functionality derived from the telogen. Two thiol telogens have been selected for study, namely 1-dodecanethiol (DDT, 4) and 1-thioglycerol (TG, 5). These offer the opportunity to study the impact of hydrophobic and hydrophilic pendant functional groups on the behaviour of the respective polymers.

The TBRT of BMEMA, using DDT as the sole telogen and AIBN (1.5 mol% based on vinyl groups) as the radical initiator, was conducted in ethyl acetate (70 °C and 50 wt% solids) with varying [BMEMA]_0_ : [DDT]_0_ molar ratios from 0.65 to 0.95. As with our previous reports of TBRT, triple detection size exclusion chromatography (TD-SEC) showed an increase in weight average molecular weight (*M*_w_) and number average molecular weight (*M*_n_) as the [MVT]_0_ : [DDT]_0_ ratio approached unity; a gel was seen at values above 0.95.

Characterisation of the crude polymerisation reactions showed vinyl group conversions ranging from 95% to >99%, as determined by ^1^H nuclear magnetic resonance (NMR) of the crude reaction mixtures, Table 1 (ESI Fig. S5–S10†). NMR studies were also used to characterise the composition of the final purified p(DDT–BMEMA) products and showed BMEMA : telogen ratios varying from 1 : 1 through to 1.12 : 1 across the series and a general trend towards higher BMEMA relative to DDT within the polymers formed at high MVT : DDT ratios (ESI Fig. S8 and S9†). This is consistent with previous reports of TBRT where MVT : DDT ratios >1 : 1 in the final products would suggest a minor degree of cyclisation.

**Table 1:** Characterisation of branched homopolymers and mixed telogen statistical copolymers synthesised *via* TBRT

	^1^H NMR				TD-SEC			
	[BMEMA]_0_ : [telogen]_0_ (*t*_0_)	Vinyl conv. (crude; %)	[BMEMA] : [telogen] (final product)	TG : DDT ratio	*M*_n_ (kg mol^−1^)	*M*_w_ (kg mol^−1^)	*Đ*	*α*
**Target polymer**								
p(DDT–BMEMA)	0.65	97	1 : 1	—	7.2	14.5	2.03	0.377
	0.71	>99	1.04 : 1	—	5.8	16.1	2.78	0.354
	0.75	95	1.07 : 1	—	8.3	15.8	1.90	0.350
	0.80	>99	1.01 : 1	—	7.8	27.5	3.51	0.333
	0.88	99	1.04 : 1	—	8.7	33.0	3.82	0.305
	0.95	96	1.12 : 1	—	9.6	28.3	2.94	0.326
	1.02	Gel	—	—	—	—	—	—
p(TG–BMEMA)	0.70	>99	—	—	15.1	58.7	3.89	0.356

**Statistical copolymers**								
p([TG–BMEMA]_0.5_-*stat*-[DDT–BMEMA]_0.5_)	0.70	>99	1.05 : 1	68 : 32	24.5	36.0	1.47	0.439
p([TG–BMEMA]_0.4_-*stat*-[DDT–BMEMA]_0.6_)	0.70	98	0.89 : 1	62 : 38	8.1	16.2	1.99	0.303
p([TG–BMEMA]_0.3_-*stat*-[DDT–BMEMA]_0.7_)	0.70	>99	0.93 : 1	54 : 46	7.5	19.3	2.57	0.280
p([TG–BMEMA]_0.2_-*stat*-[DDT–BMEMA]_0.8_)	0.70	>99	0.81 : 1	50 : 50	10.7	18.7	1.75	0.375

TBRT was repeated using TG as the sole telogen to prepare p(TG–BMEMA) and introduce hydrophilic side groups onto the polyester-amine branched architecture. The synthesis was conducted at a [BMEMA]_0_ : [TG]_0_ molar ratio of 0.7, and fully soluble products were obtained with >99% vinyl group conversion, Table 1.

As mentioned previously, mixed telogens provide an opportunity to form statistical copolymer structures using TBRT strategies. TG and DDT were combined into a single BMEMA polymerisation at ratios from 50 : 50 mol% through to 20 : 80 mol% (TG : DDT), leading to p([TG–BMEMA]_*x*_-*stat*-[DDT–BMEMA]_*y*_) branched polyester-amines with a mixture of hydrophobic straight-chain alkyl and hydrophilic diol pendant groups, 6 (Fig. 1B). After purification, the resulting polymers were characterised in detail, and ^1^H NMR analysis determined the relative incorporation of the two telogens as being weighted towards TG, Table 1 (ESI Fig. S11–S13†); the higher than targeted amounts of TG within the final polymers may be due to removal of polymeric species containing higher concentrations of DDT during purification, or a more rapid rate of incorporation of TG due to variable chain transfer rates. This is currently being studied in more detail.

The impact of varying telogen incorporation was readily observed in simple aqueous solubility studies under varying pH conditions (Fig. 1C and D). Addition of deionised water (10 mL) to p(DDT–BMEMA) (*M*_w_ = 33.0 kg mol^−1^) led to no discernible dissolution and observable undissolved material within the vial (polymer concentration = 3 mg mL^−1^; Fig. 1C(i) and (iv)). When the samples of p(TG–BMEMA) (*M*_w_ = 58.7 kg mol^−1^) and p([TG–BMEMA]_0.5_-*stat*-[DDT–BMEMA]_0.5_) (*M*_w_ = 36.0 kg mol^−1^) were subjected to the same procedure, slightly opaque solutions were observed with complete disappearance of the sample (Fig. 1C(ii) and (iii)). Addition of a single drop of 1 M HCl to each sample (pH ∼ 5) led to considerable and immediate changes. First, p(DDT–BMEMA) formed an opaque dispersion with a small residual undispersed sample still visible (Fig. 1D(i)); however, the TG-containing branched polyester-amines became completely transparent (Fig. 1D(ii) and (iii)), indicating full solubilisation. Upon shaking, the p(TG–BMEMA) sample rapidly settled to a clear solution, whereas p([TG–BMEMA]_0.5_-*stat*-[DDT–BMEMA]_0.5_) clearly demonstrated its amphiphilic and surface-active character through the formation of a persistent foam (Fig. 1D(ii) and (iv)). Interestingly, shaking of the p(DDT–BMEMA) sample did not lead to full dissolution, but a low level of foaming was also seen, also indicating an amphiphilic nature of this polymer, presumably from the combination of protonated backbone amines and the pendant hydrophilic C_12_ alkyl chains.

In addition to the statistical mixed telogen copolymers described above (Fig. 1B), we also synthesised a statistical mixed mono-vinyl taxogen:MVT copolymer^44^ (Fig. 2) to evaluate the importance of backbone and pendant tertiary amines for the pH stimuli response of TBRT polymers. The MVT ethylene glycol dimethacrylate (EGDMA, 7) and the mono-vinyl taxogen 2-(diethylamino)ethyl methacrylate (DEAEMA, 8) were therefore polymerised under TBRT conditions in toluene. An [EGDMA]_0_ : [DEAEMA]_0_ molar ratio of 1 : 1 was employed, and [MVT]_0_ : [DDT]_0_ ratios were varied to establish reaction conditions that would generate soluble, branched polymers at high vinyl group conversion. For clarity, the resulting purified p(DDT–EGDMA–DEAEMA) polymers are branched polyesters and contain no backbone tertiary amines but rather pendant amino functionality, 9 (Fig. 2). The structural complexity of p(DDT–EGDMA–DEAEMA) is worth noting as the mono-vinyl taxogen is incorporated into the polymerisation of the MVT in a statistical manner which leads to a near identical ratio of tertiary amines and vinyl group residues as TBRT polymers derived from BMEMA (10 = nominal repeat unit structure), but the distribution would not be expected to be homogeneous. BMEMA leads to regularly spaced amine functionality within the backbone of the branched polymers, but the inclusion of DEAEMA may lead to ‘mid chain amines’, where the pendant amine lies within a telomer, an ‘end group amine’, where the pendant amine is the first monomer that the thiyl radical reacts with, but propagation occurs with other MVT units, or a ‘modified DP_1_’ group, where the reaction of the thiyl radical only involves one MVT vinyl group and one DEAEMA monomer, 9 (Fig. 2). Other structures are also possible, and it is plausible to expect that some telomer structures within the branched architecture do not bear amine groups.

The synthesis of p(DDT–EGDMA–DEAEMA) was studied in an identical strategy to that employed for the TBRT of BMEMA through maintaining a 1 : 1 EGDMA : DEAEMA ratio but varying the DDT content within the reaction relative to EGDMA, at 50 wt% solids in toluene, Table 2. As discussed in previous reports, under ideal conditions, the incorporation of a mono-vinyl monomer would allow a longer number average chain length for the telomer distribution as some of the propagating vinyl groups do not contribute to branching. Steadily increasing *M*_w_ values were observed with decreasing DDT content; however, at higher concentrations of DDT, relatively few DEAEMA residues were observed in the purified polymer samples. The DEAEMA content also increased with decreasing DDT, suggesting a potential impact of varying reactivity ratios on this TBRT statistical copolymer synthesis and removal of DEAEMA-rich species during purification, Table 2 (ESI Fig. S14–S19†). Note: at an [EGDMA]_0_ : [DDT]_0_ value of 0.92, the onset of microgelation appears to have impacted this trend with sudden increases in both *M*_n_ and *M*_w_.

**Table 2:** Characterisation of mixed mono-vinyl/multi-vinyl taxogen statistical copolymers synthesised *via* TBRT

^1^H NMR			TD-SEC			
[EGDMA]_0_ : [DDT]_0_ (*t*_0_)	Vinyl conv. (crude; %)	[EGDMA] : [DEAEMA] : [DDT] (final product)	*M*_n_ (kg mol^−1^)	*M*_w_ (kg mol^−1^)	*Đ*	*α*
0.59	>99	1 : 0.19 : 1	2.6	16.1	6.28	0.291
0.75	>99	1 : 0.44 : 1	4.1	49.7	12.25	0.230
0.80	97	1 : 0.63 : 1	3.7	71.2	19.46	0.321
0.88	>99	1 : 0.70 : 1	4.2	210.5	50.26	0.339
0.92	>99	1 : 0.35 : 1	22.9	571.2	24.97	0.346
0.98	>99	Gel	—	—	—	—

The mixed mono-vinyl taxogen/MVT statistical copolymer p(DDT–EGDMA–DEAEMA) synthesised at an EGDMA : DDT molar ratio of 0.80 has approximately one tertiary amine per 1.6 EGDMA units and a relatively low *M*_w_ (71.2 kg mol^−1^) compared to two other samples synthesised at lower starting DDT concentrations within the TBRT reaction. This polymer was selected to compare against the targeted statistical telogen copolymer p([TG–BMEMA]_0.3_-*stat*-[DDT–BMEMA]_0.7_), which contains a measured TG : DDT molar ratio of 54 : 46, in terms of their behaviour under nanoprecipitation conditions. This statistical telogen copolymer was selected first due to the MVT : telogen ratio within the final product being close to unity, but also as the TG : DDT ratio was close to 50 : 50, thereby allowing a good balance of hydrophobic and hydrophilic telogen-derived pendant groups.

The two copolymers were readily soluble in THF (5 mg mL^−1^), and after rolling for 24 hours, 1 mL of each solution was dripped into a rapidly stirred volume of deionised water (5 mL) under ambient conditions. As in our previous reports, the open vials were left to stir in a laminar flow cabinet at ambient temperature for 48 hours to allow evaporation of THF, and regular addition of water ensured a final dispersion containing the precipitated nanoparticles at 1 mg mL^−1^ (for completeness, p(TG–BMEMA) and p(DDT–BMEMA) were unable to successfully form nanoparticles under these conditions).

**Fig. 3:**
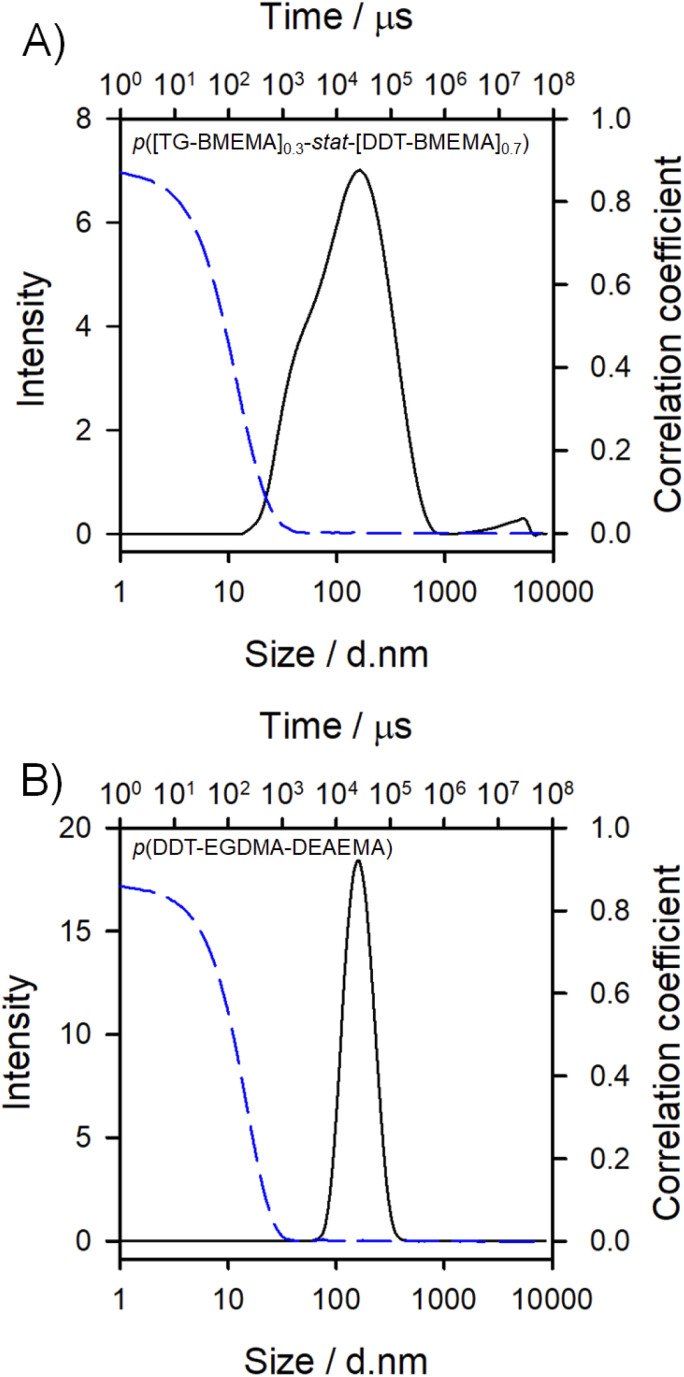
Dynamic light scattering analysis of nanoprecipitations using branched TBRT polymers. Overlaid intensity distributions (solid black lines) and correlograms (dotted blue lines) of (A) nanoprecipitated mixed telogen statistical copolymer p([TG–BMEMA]_0.3_-*stat*-[DDT–BMEMA]_0.7_), and (B) nanoprecipitated mixed mono-vinyl/multi-vinyl taxogen copolymer p(DDT–EGDMA–DEAEMA). The samples shown are 1 mg mL^−1^ in water and are unfiltered.

Dynamic light scattering (DLS) analysis of the final nanoparticle dispersions confirmed successful nanoprecipitation; however, p([TG–BMEMA]_0.3_-*stat*-[DDT–BMEMA]_0.7_) generated a broad and bimodal distribution of particles with a *z*-average diameter (*D*_*z*_) of approximately 100 nm, a polydispersity index (PDI) of 0.413, and a zeta potential (*ζ*) of 44.8 mV. In contrast, p(DDT–EGDMA–DEAEMA) generated a narrow distribution of nanoparticles (*D*_*z*_ = 155 nm, PDI = 0.072, and *ζ* = 32.8 mV; Fig. 3).

The positioning of the amine groups, either in the branched polyester-amine backbone for p([TG–BMEMA]_0.3_-*stat*-[DDT–BMEMA]_0.7_) (Fig. 3A), or as pendant groups to the branched polyester in p(DDT–EGDMA–DEAEMA), appeared to have no impact on the formation of considerable positive zeta potential values, indicating the accessibility of the amines for protonation in both cases. The response of the nanoparticles to changes in pH was therefore of considerable interest, and studies were conducted using an autotitrator to slowly add HCl (0.12 M) with continuous monitoring by DLS.

**Fig. 4:**
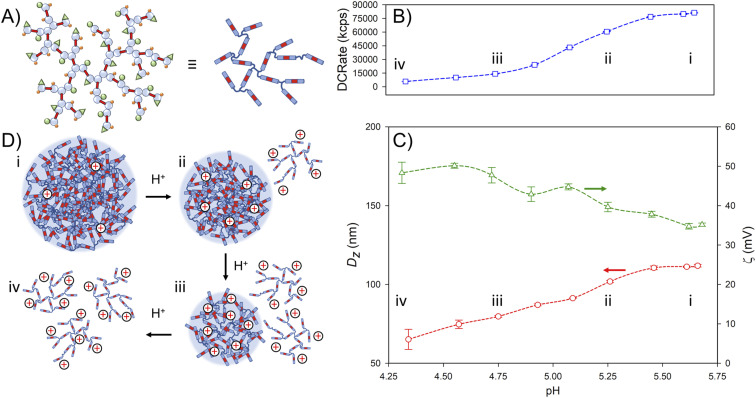
Schematic representation and dynamic light scattering data from studies of the impact of HCl on aqueous dispersions of TBRT polymer nanoprecipitates. (A) Representations of the mixed telogen statistical copolymer p([TG–BMEMA]_0.3_-*stat*-[DDT–BMEMA]_0.7_); (B) impact on the derived count rate (DCRate) as the pH of the aqueous nanoprecipitate environment is decreased by acid addition (experiment progresses from right to left); (C) subsequent impact of decreasing pH on the observed nanoprecipitate *z*-average diameter (red circles) and zeta potential (green triangles); and (D) schematic description of particle behaviour with increasing acid addition: (i) initial nanoparticles under natural pH after the nanoprecipitation procedure (corresponds to B(i) and C(i)); (ii) increased protonation leading to dissolution of some branched polymer chains (corresponds to B(ii) and C(ii)); (iii) further dissolution after increased acid addition (corresponds to B(iii) and C(iii)), and (iv) full dissolution of the TBRT copolymer (corresponds to B(iv) and C(iv)).

The nanoparticles formed from p([TG–BMEMA]_0.3_-*stat*-[DDT–BMEMA]_0.7_) exhibited very little change in the derived count rate (DCRate) and *D*_*z*_ during the initial addition of acid, to a measured pH of 5.46, with a concomitant increase in *ζ* (Fig. 4B(i) and C(i)). From this point, further addition of acid led to a dramatic decrease in the derived count rate, decreasing *D*_*z*_ values and increasing *ζ* (Fig. 4B(ii) and C(ii)). At a pH value of 4.75, the nanoprecipitates decreased in *D*_*z*_ by approximately 30%, the derived count rate had reduced by >80% and the measured *ζ* values had increased by approximately 35% (Fig. 4B(iii) and C(iii)). At this pH value, the data collected by DLS were no longer of high quality; however, the titration was continued to provide a view of subsequent changes. As can be seen in Fig. 4B(iv) and C(iv), the derived count rate drops to approximately 5% of its original value at a pH value of 4.34, and *ζ* values continue to increase with added acid.

The data suggest a steady protonation of the backbone amine groups present within the nanoparticles (Fig. 4D(i)), followed by a steady solubilisation of the branched polymer chains, a decrease in nanoparticle size, and the formation of a polymer solution (Fig. 4D(ii)–(iv)).

**Fig. 5:**
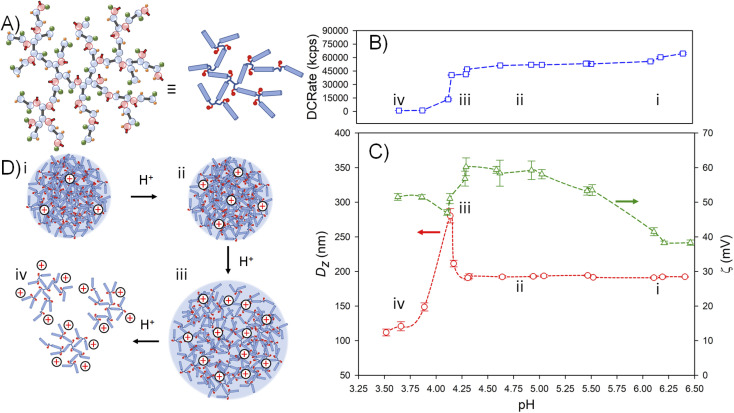
Schematic representation and dynamic light scattering data from studies of the impact of HCl on aqueous dispersions of TBRT polymer nanoprecipitates. (A) Representations of the mixed mono-vinyl/multi-vinyl taxogen statistical copolymer p(DDT–EGDMA–DEAEMA); (B) impact on the derived count rate (DCRate) as pH of the aqueous nanoprecipitate environment is decreased by acid addition (experiment progresses from right to left); (C) subsequent impact of decreasing pH on the observed nanoprecipitate *z*-average diameter (red circles) and zeta potential (green triangles); and (D) schematic description of particle behaviour with increasing acid addition: (i) initial nanoparticles under natural pH after the nanoprecipitation procedure (corresponds to B(i) and C(i)); (ii) increased protonation leading to no observable diameter increase but a clear increase in the zeta potential (corresponds to B(ii) and C(ii)); (iii) further protonation leading to considerable swelling of the particles (corresponds to B(iii) and C(iii)), and (iv) full dissolution of the TBRT copolymer (corresponds to B(iv) and C(iv)).

Titration of the nanoprecipitates formed from p(DDT–EGDMA–DEAEMA) showed very different behaviour. The initial addition of acid also led to no appreciable changes in the derived count rate (−6%), *D*_*z*_ (no change) or *ζ* (no change) at a pH value of 6.20 (Fig. 5B(i) and C(i)). At this point, further addition of acid led to a noticeable increase in *ζ* values but only negligible changes in both the derived count rate and *D*_*z*_ (Fig. 5B(ii) and C(ii)). At a pH value of 4.64, corresponding to a value below the loss of meaningful data from the titration of p([TG–BMEMA]_0.3_-*stat*-[DDT–BMEMA]_0.7_), the *D*_*z*_ value still exhibited no change, and the derived count rate had reduced by only 20%, but the *ζ* values had increased by a factor of >1.5.

No further appreciable changes were noted until a pH value of approximately 4.25 when a sudden increase in *D*_*z*_ and a simultaneous decrease in the derived count rate and *ζ* were seen (Fig. 5B(iii) and C(iii)), reaching a maximum *D*_*z*_ value at pH = 4.14. Further addition of acid led to a considerable decrease in all observed values with a limit of data quality seen at a pH = 3.89 (Fig. 5B(iv) and C(iv)).

The DLS data suggest that the placement of pendant amines allows the hydrophobic backbone of p(DDT–EGDMA–DEAEMA) to resist solubilisation during increasing protonation (Fig. 5D(i) and D(ii)). This is in stark contrast to the behaviour of p([TG–BMEMA]_0.3_-*stat*-[DDT–BMEMA]_0.7_) where protonation appears to directly impact the hydrophilicity of the branched polymer ester-amine backbone and dissolution of the particles. The p(DDT–EGDMA–DEAEMA) nanoparticles are remarkably stable to protonation, when compared to p([TG–BMEMA]_0.3_-*stat*-[DDT–BMEMA]_0.7_), reaching *ζ* > 60 mV before swelling by nearly 150% (Fig. 5D(iii)) and undergoing a very sharp breakdown and dissolution (Fig. 5D(iv)). The impact of the presence of TG as an additional pendant group that aids overall hydrophilicity is also important to consider. In the absence of TG, nanoprecipitation was not observed for p(DDT–BMEMA), and successful nanoprecipitation is critical to this comparison. TG is not pH-sensitive and is not expected to have a considerable impact on the pH response of the nanoparticles *per se*, but it does enable the hydrophilicity of the protonated TBRT polymers, as seen in Fig. 1. The ability of p(DDT–EGDMA–DEAEMA) to form uniform nanoprecipitates with a narrow distribution without any TG-derived side groups again underlines the impact of amine placement on the behaviour of these polymers.

As a direct comparison with p(DEAEMA), an aqueous solution of p(DDT–EGDMA–DEAEMA) was formed by addition of HCl until a clear solution with a pH = 2.5 was observed, and hydrogen ion titration was conducted *via* slow addition of 0.1 M KOH(aq). Despite the potential issues arising from the insolubility of p(DDT–EGDMA–DEAEMA) at relatively low pH values (Fig. 5), the titration followed the behaviour of other amine-containing polymers and allowed a ready determination of p*K*_a_ (ESI Fig. S20†). p(DEAEMA) has a reported p*K*_a_ = 7.3 (ref. 47) in similar studies; however, the p*K*_a_ of p(DDT–EGDMA–DEAEMA) was found to be 5.3. This value is significantly below that of p(DEAEMA), but within the reported range of tertiary amine methacrylates. The TBRT statistical copolymers shown here clearly modify the environment of the amine and its response to variations in pH. Importantly, the titration was also monitored by DLS, and a significant increase in the derived count rate was observed upon KOH addition, with visible turbidity apparent at a pH of approximately 4.1 (ESI Fig. S20†). This correlates almost exactly with the sudden loss of the derived count rate and dissolution of the nanoprecipitates upon the addition of HCl (Fig. 5B and ESI Fig. S21†) and suggests pH-responsive precipitation and aggregation with increasing pH that mirrors the disassembly and dissolution response of the nanoprecipitates with decreasing pH.

## Conclusions

TBRT offers access to new polymer architectures with backbone chemistries that would be normally associated with step-growth chemistries. Here, we have utilised TBRT to allow comparison of branched nanoprecipitates formed from polyester-amine or polyester backbones with pendant amine groups. The pH response is markedly different, but the synthesis of the polymers was relatively facile given the use of conventional free radical approaches within TBRT. This opens many avenues of future investigation as the flexibility of TBRT allows considerable chemical and architectural variation through selection of commercially available vinyl monomers (taxogens) and thiols (telogens). As we show here, the design and synthesis of novel MVTs, mono-vinyl taxogens, and even telogens, would allow considerable opportunities to study and discover new polymers with highly novel properties. The encapsulation and stimuli-responsive release of active molecules is an area of significant global interest, and the flexibility of TBRT provides a new platform for innovation within this field.

## Author contributions

SPR was responsible for grant funding, conceptualisation of the original research programme, supervision, writing of the original draft, editing and review. PC contributed to supervision, formal analysis, validation, and manuscript review. SRC contributed to supervision/experimentation and data curation. OBPL contributed to experimentation, data curation, formal analysis, and manuscript review.

## Conflicts of interest

SPR, PC and SRC are co-inventors on patents that protect the TBRT chemistry; these patents have been licensed to Scott Bader and form the basis of Polymer Mimetics Ltd (Company number 12598928).

## Supplementary Material

NA-004-D2NA00399F-s001
