# Supplementary material for: Controlling the pH-response of branched copolymer nanoprecipitates synthesised by transfer-dominated branching radical telomerisation (TBRT) through telogen chemistry and spatial distribution of tertiary amine functionality
Source: Nanoscale Adv. 2022 Aug 22;4(19):4051–8. doi: 10.1039/d2na00399f (PMC9514558; doi:10.1039/d2na00399f)
Supplement: NA-004-D2NA00399F-s001 [file NA-004-D2NA00399F-s001.pdf]

Electronic Supplementary Material (ESI) for Nanoscale Advances.
This journal is © The Royal Society of Chemistry 2022

Controlling the pH-response of branched copolymer nanoprecipitates synthesised by Transfer-dominated Branching Telomerisation (TBRT) through telogen chemistry and spatial distribution of tertiary amine functionality.

Oliver B. Penrhyn-Lowe, Savannah R. Cassin, Pierre Chambon and Steve P. Rannard*

*Corresponding author: E-mail: srannard@liv.ac.uk

Supplementary Information:

Materials and Methods

Experimental

Supplementary Figure S1 – S19

Experimental

Materials

N-methyldiethanolamine (N-MDEA, 98%) and 1-dodecanethiol (DDT, 98%) were purchased from Alfa Aesar. Methacryloyl chloride (97%, 200 ppm MEHQ), triethylamine (> 99.5%), 2,2'-azobisisobutyronitrile (AIBN, 98%), 2-(diethylamino)ethyl methacrylate (DEAEMA, 98%, 100 ppm phenolthiazine), ethyleneglycol dimethacrylate (EGDMA, 98%, 100 ppm MEHQ), 1-thioglycerol (98%), 4-methoxy phenol (MEHQ, 99%), chloroform-d ($CDCl_3$, 99.8 atom% D) and aluminium oxide (activated, neutral, Brockmann I) were purchased from Sigma Aldrich. Sodium sulfate (anhydrous, 99%) was purchased from Acros Organics. Sodium hydrogen carbonate (99.7%), diethyl ether, methanol, toluene, petroleum ether, tetrahydrofuran and ethyl acetate were purchased from Fischer. All materials were used as received unless otherwise stated.

Methods

All NMR spectra were recorded using a Bruker Advance DPX spectrometer operating at 400 MHz, with all chemical shifts (δ) reported in parts per million (ppm) and scalar couplings in Hertz (Hz). TD-SEC data was obtained using a Malvern Viscotek instrument. One instrument was fitted with a GPCmax VE2001 auto-sampler, two Viscotek T6000 columns (and a guard column), a VE3580 refractive index detector and a Viscotek 270 Dual Detector (viscometer and light scattering) with a mobile phase of THF containing 2 v/v % trimethylamine, utilised at a flow rate of 1 mL min^{-1} and at 35 °C. Polystyrene standards, at 105 kDa and 245 kDa, were utilised as narrow and broad standards, respectively. The second instrument was equipped with a GPCmax VE2001 auto-sampler, two Viscotek T6000 columns (and a guard column) and a triple detector array TDA305 (refractive index, viscometer and light scattering) with a mobile phase of DMF containing 0.01 M lithium bromide, utilised at a flow rate of 1 mL min-1 and at 60 °C. Poly(methyl methacrylate) standards, at 60 kDa and 95 kDa, were utilised as narrow and broad standards, respectively. IR spectra were obtained using a Bruker Alpha FTIR instrument. Mass Spectrometry data was obtained using a Micromass LCT Mass Spectrometer operating in Electrospray Ionisation (ESI) mode. Nanoparticle size and zeta potential data were obtained by Dynamic Light Scattering (DLS) measurements using a Zetasizer Nano ZS instrument equipped with a detector at 173° (backscatter direction) and using Malvern DTS1070 cuvettes. Auto-titration was controlled using a Malvern MPT-2 multi-purpose titrator, using hydrochloric acid (0.11975 M and 0.011975 M) and sodium hydroxide (0.0896

M) stock solutions, using a Malvern DTS1070 cuvette. The titration programme was conducted autonomously through the Zetasizer software.

Sample preparation

All NMR samples were dissolved in either $CDCl_3$ or DMSO-d_6 and filtered. ^{13}C, Correlation Spectroscopy (HH-COSY), Heteronuclear Single-Quantum Coherence (HSQC) and Attached Proton Test (APT) experiments were run at ≥ 50 mg mL^{-1}. TD-SEC samples were dissolved in either THF (2 v/v % TEA) or DMF (0.01 M LiBr) solvent at 10 mg mL^{-1} and homogenised for 24 hours before being filtered through a 0.2 μm syringe. Nanoprecipitations were conducted by dispersing a solution of polymer (5 mg mL^{-1} in THF) in a rapidly stirring aqueous phase (5 mL), before allowing the THF to evaporate off with continued stirring over 48 hours.

Synthesis of N,N-bis(methacryloxyethyl) methylamine (BMEMA)

A two-necked round bottomed flask, equipped with a stirrer bar, was loaded with diethyl ether (20 mL), triethylamine (40 mL, 0.25 mol, 3 eqv), and N-methyldiethanolamine (10 mL, 83.9 mmol, 1 eqv). A dropping funnel was applied to the flask and loaded with diethyl ether (40 mL) and methacryloyl chloride (21 mL, 0.21 mol, 2.5 eqv). The other neck was plugged with a septa and needle to prevent a pressure increase. Dropwise addition from the funnel, with continual stirring, was conducted in an ice bath over 30 minutes. The resultant mixture was stirred at room temperature for a further 24 hrs and the precipitate was filtered through fluted paper. The organic layer was washed with saturated sodium hydrogen carbonate (3 x 100 mL) and water (2 x 100 mL) and was then dried over sodium sulphate and filtered thereafter. 4-methoxyphenol (50 mg, 4800 ppm) was added as an inhibitor to the solution before evaporation of the solvent under vacuum at ambient temperature, yielding the product as a clear orange liquid (18.8581 g, 88%). (Found: FTIR (ν_{max}/cm^{-1}) 1714 (C=O), 1637 (C=C), 1153 (C-O), 1018 (C-N); δH (400 MHz; $CDCl_3$; Me_4Si): 6.08 (2 H, s, H_{cis}HC=C-CO), 5.54 (2 H, s, H_{trans}HC=C-CO), 4.23 (4 H, t, J = 5.9, O-CH_2-), 2.77 (4 H, t, J = 5.9, N-CH_2-), 2.36 (3 H, s, N-CH_3), 1.92 (6 H, s, C=C-CH_3); δC (400 MHz; $CDCl_3$; Me_4Si): 167.34, 136.25, 125.54, 62.69, 55.93, 43.01, 18.30); m/z (ESI) 256.1547 $(M+H)^+$, $C_{13}H_{21}NO_4$ requires 255.15); CHN: C 60.1, H 8.0, N 4.7, $C_{13}H_{21}NO_4$ requires C 61.1, H 8.2, N 5.5%)

TBRT of N,N-bis(methacryloxyethyl) methylamine (BMEMA) with DDT

In a typical experiment targeting a $[BMEMA]_0/[DDT]_0$ ratio of 0.80, a 10 mL round-bottomed flask, equipped with a stirrer bar, was loaded with BMEMA (0.50 g, 1.96 mmol, 0.80 eqv),

DDT (0.49 g, 2.45 mmol, 1.00 eqv), AIBN (9.6 mg, 58.75 μmol, 1.5 mol% *vs* vinyl bonds), and EtOAc (0.99 g, 11.30 mmol, 50 wt% *vs* BMEMA + DDT). The solution was homogenised by agitation and a sample was extracted for ^{1}H NMR analysis. The solution was deoxygenated using a nitrogen purge for a minimum of 20 minutes, and was then heated to 70 ˚C and allowed to proceed for 24 hours with continual stirring. The reaction was ceased by exposure to air and cooling to ambient temperature. The crude reaction mixture was then precipitated into stirring methanol (50 mL) at 0 ˚C, affording a tacky yellow solid. The product was washed further with fresh cold methanol (3 × 20 mL) and was dried *in vacuo* at 40 ˚C for 16 hours.

Specific molar ratios of BMEMA/DDT can be found in Table 1.

TBRT of BMEMA with TG and DDT

In an experiment targeting a 1:1 incorporation of DDT:TG, a 10 mL round-bottomed flask, equipped with a stirrer bar, was loaded with BMEMA (0.50 g, 1.96 mmol, 0.70 eqv), DDT (0.28 g, 1.40 mmol, 0.71 eqv), TG (0.15 g, 1.40 mmol, 0.71 eqv), AIBN (9.6 mg, 58.75 μmol, 1.5 mol% *vs* vinyl bonds), and EtOAc (0.93 g, 10.61 mmol, 50 wt% *vs* BMEMA + DDT + TG). The solution was homogenised by agitation and a sample was extracted for ^{1}H NMR analysis. The solution was deoxygenated using a nitrogen purge for a minimum of 20 minutes, and was then heated to 70 ˚C and allowed to proceed for 24 hours with continual stirring. The reaction was ceased by exposure to air and cooling to ambient temperature. The crude reaction mixture was then precipitated into stirring petroleum ether (b.p. 40-60 ˚C, 50 mL) at 0 ˚C, affording a tacky yellow solid. The product was washed further with fresh cold petroleum ether (3 × 20 mL) and was dried *in vacuo* at 40 ˚C for 16 hours.

Specific molar ratios for all statistical mixed-telogen copolymers of BMEMA with DDT and TG can be found in Table 1.

TBRT of ethylene glycol dimethacrylate (EGDMA) and 2-(diethylamino)ethyl methacrylate (DEAEMA) with DDT

In a typical experiment targeting an $[EGDMA]_0/[DDT]_0$ ratio of 0.80, a 10 mL round-bottomed flask, equipped with a stirrer bar, was loaded with EGDMA (0.80 g, 4.04 mmol, 0.80 eqv), DEAEMA (0.75 g, 4.04 mmol, 0.80 eqv), DDT (1.02 g, 5.05 mmol, 1.00 eqv), AIBN (29.8 mg, 0.18 mmol, 1.5 mol% *vs* vinyl bonds), and toluene (2.57 g, 27.88 mmol, 50 wt% *vs* EGDMA + DEAEMA + DDT). The solution was homogenised by agitation and a sample was extracted for ^{1}H NMR analysis. The solution was deoxygenated using a nitrogen purge for a

minimum of 20 minutes, and was then heated to 70 ˚C and allowed to proceed for 24 hours with continual stirring. The reaction was ceased by exposure to air and cooling to ambient temperature. Toluene was removed *in* vacuo and the crude mixture solubilised in THF (5 mL) before being precipitated into stirring methanol (100 mL) at 0 ˚C, affording a white solid. The product was washed further with fresh cold methanol (3 × 50 mL) and was dried *in vacuo* at 40 ˚C for 16 hours.

Specific molar ratios of EGDMA/DDT can be found in Table 2. All reactions were conducted at an EGDMA:DEAEMA molar ratio of 1:1.

Nanoprecipitation of statistical copolymers

A sample of appropriate polymer was solubilised in THF (5 mg mL^{-1}) and homogenised for 24 hours. The polymer solution (1 mL) was injected into rapidly stirring deionised water (5 mL). The mixture was then left stirring for 48 hours, allowing the THF to evaporate. Any mass loss associated with the evaporation was water was accounted for by further addition of deionised water, yielding nanoparticle dispersions at concentrations of 1 mg mL^{-1}.

Hydrogen ion titration

The mixed mono-vinyl/multi-vinyl taxogen statistical copolymer *p*(DDT-EGDMA-DEAEMA), synthesised at an EGDMA/DDT molar ratio of 0.80, was dissolved in deionised water and adjusted to pH≈2.5 using HCl in order to obtain a solution containing approximately 1.5×10^{-2} mol L^{-1} of tertiary amine residues. The titration curve, Figure S20, was obtained by monitoring the pH as a function of the volume of KOH 0.1 M added. The derived count rate of the aqueous medium was monitored *via* dynamic light scattering (DLS, Zetasizer nano-S). Samples were taken every 1 mL of KOH solution added, until visible aggregation occurred, and analysed by setting the measurement position and attenuator values of the DLS instrument to 4.65 mm and 7, respectively. After each measurement the samples were returned to the main titration beaker and a new volume of KOH was added.

The pH was monitored using a Hanna Instruments pH meter HI2211 fitted with a HI1131 probe and calibrated using buffer solutions at pH = 7.00 and pH = 4.01.

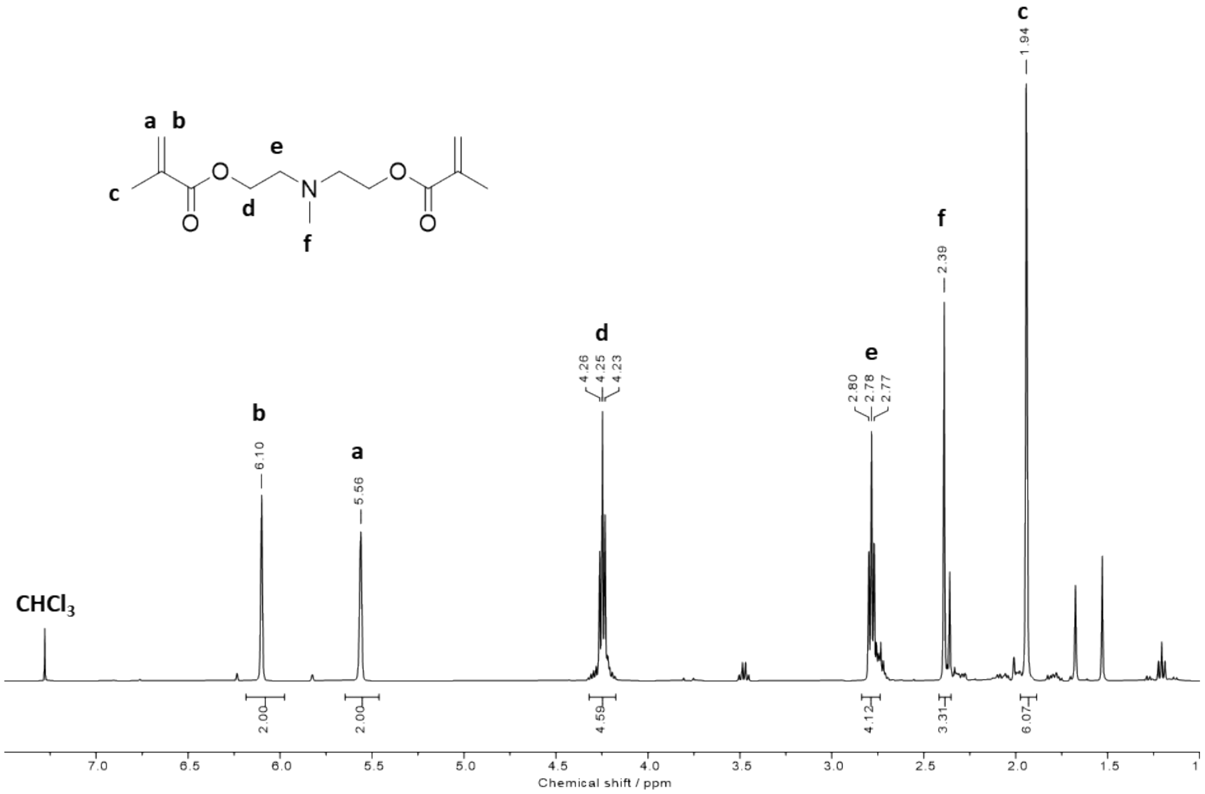

Figure S1 ^{1}H NMR analysis of N,N-bis(methacryloxyethyl) methylamine.

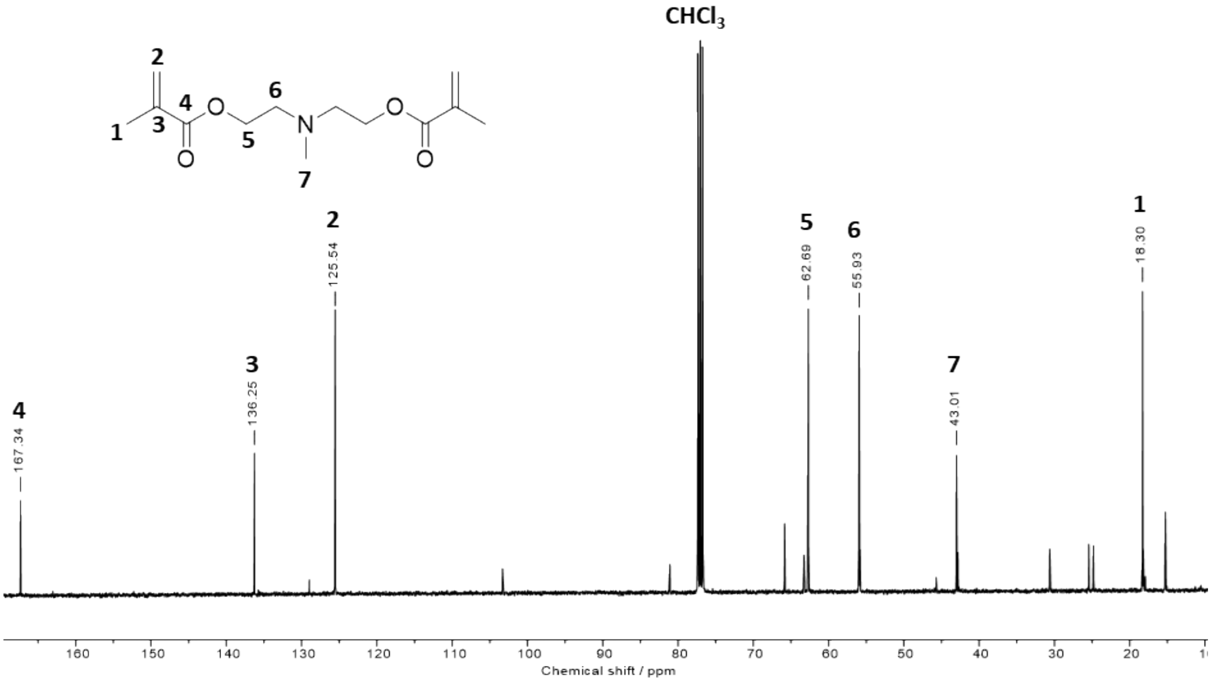

Figure S2 ^{13}C NMR analysis of N,N-bis(methacryloxyethyl) methylamine.

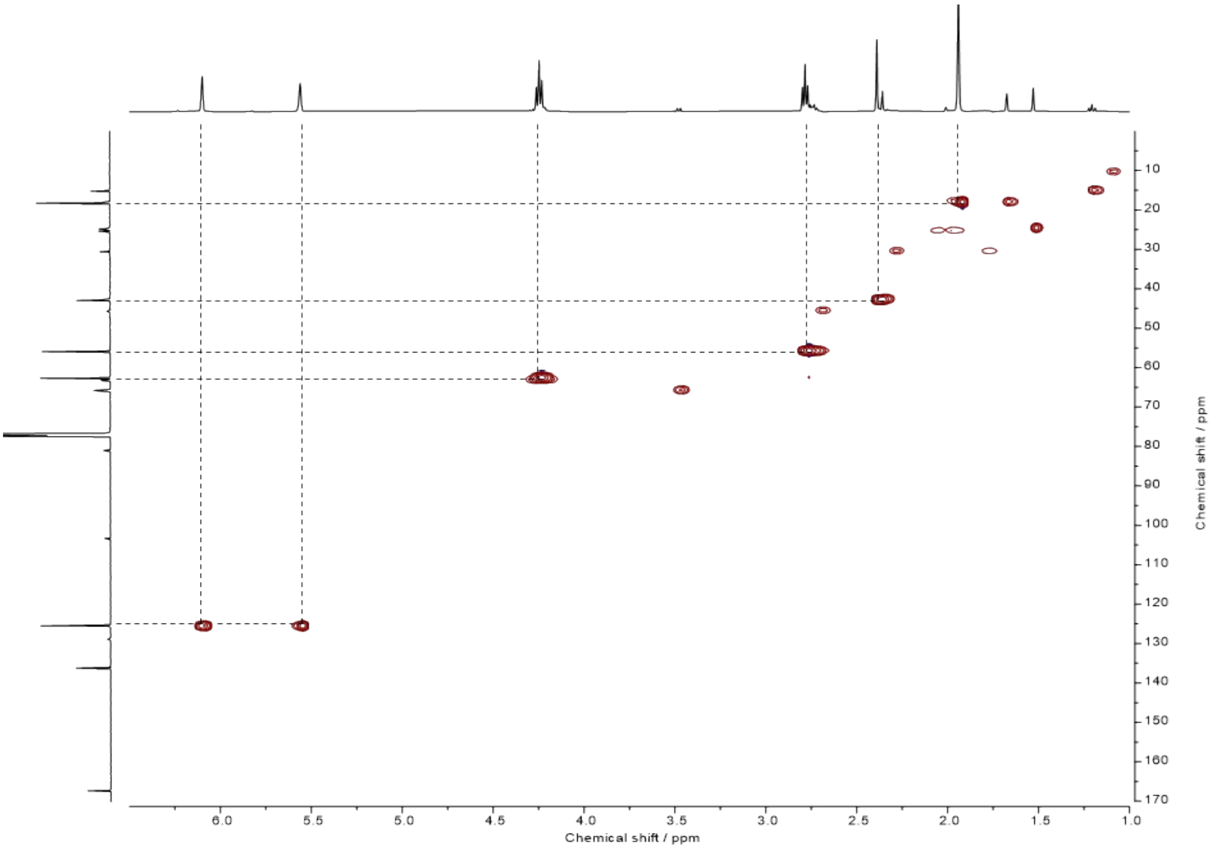

Figure S3 $^{1}H^{13}C$ HSQC NMR analysis of N,N-bis(methacryloxyethyl) methylamine.

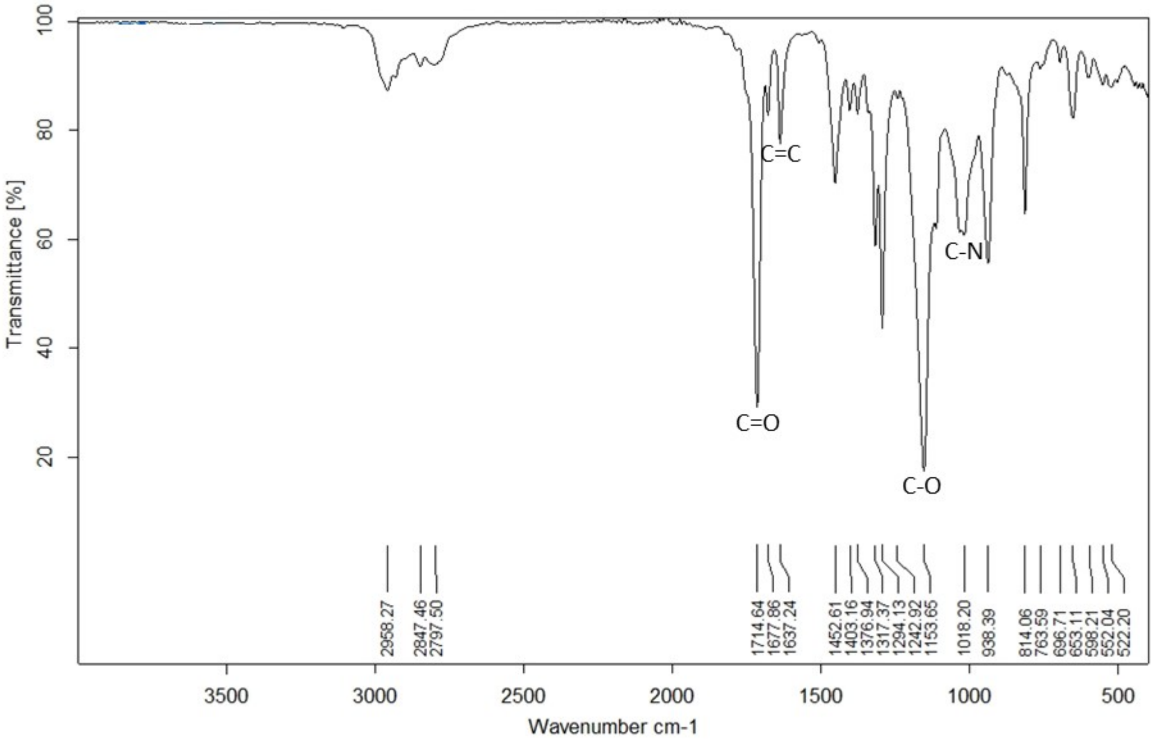

Figure S4 FTIR spectrum of N,N-bis(methacryloxyethyl) methylamine.

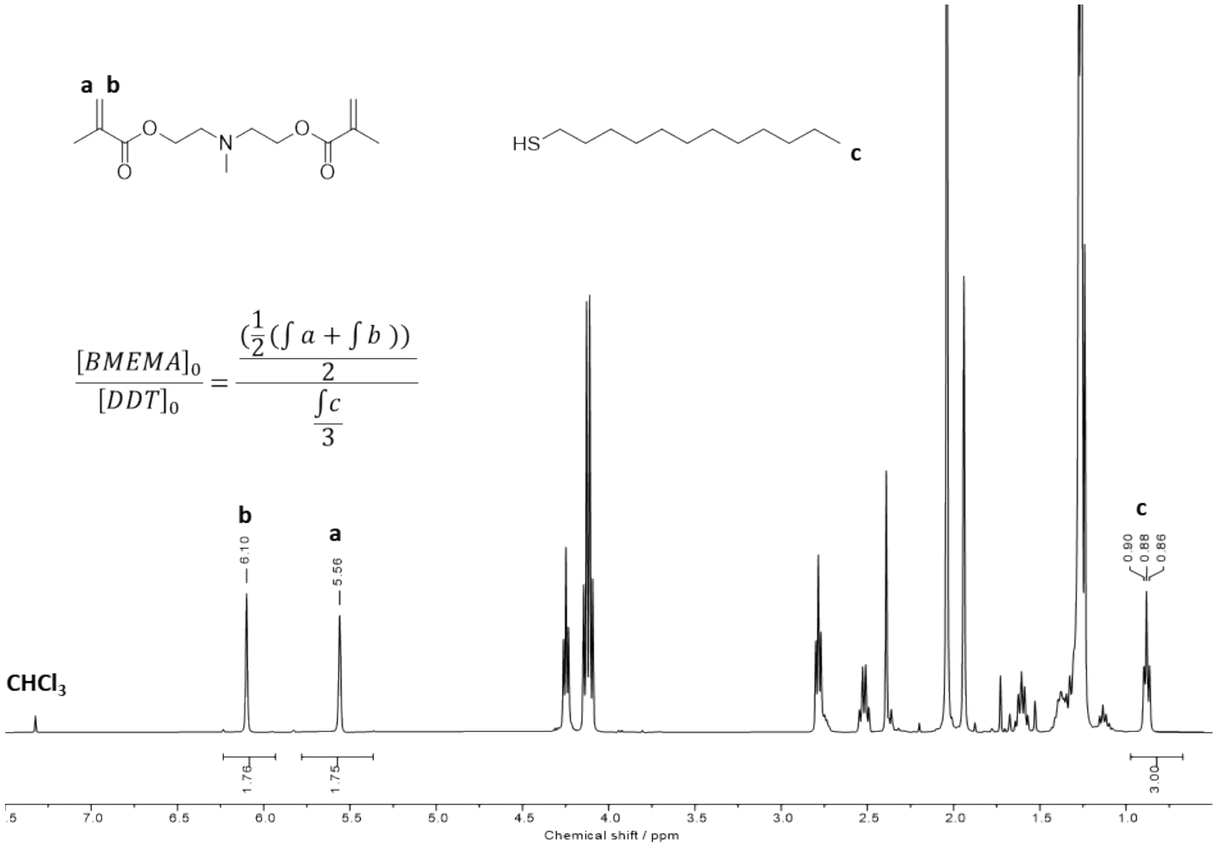

Figure S5 ^{1}H NMR analysis taken at t_0 for the TBRT of BMEMA and DDT, targeting a $[BMEMA]_0/[DDT]_0$ ratio of 0.88 (Table 1).

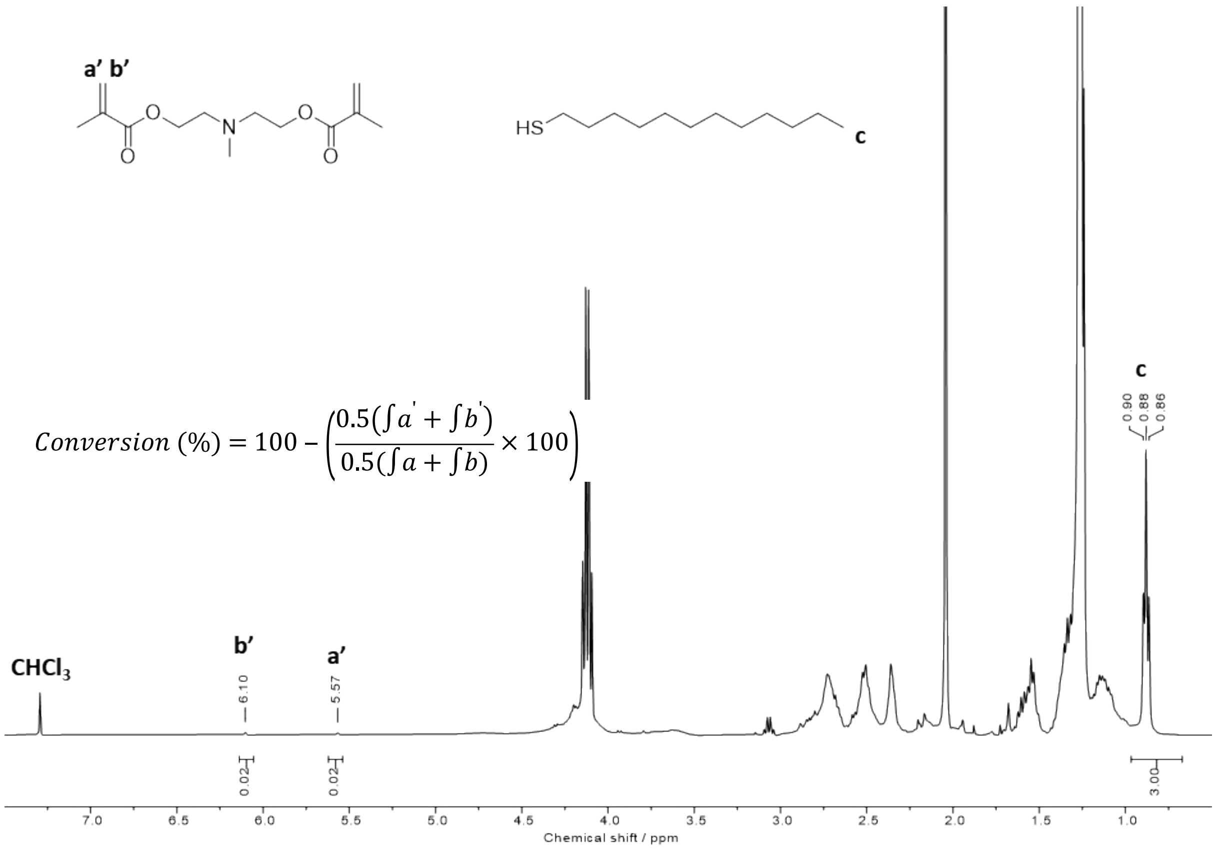

Figure S6 ^{1}H NMR analysis of the crude product for the TBRT of $[BMEMA]_{0.88}[DDT]_{1.00}$, showing an example calculation for vinyl bond conversion referenced against analysis conducted at t_0. Signal **c** is utilised as a common integral calibrant.

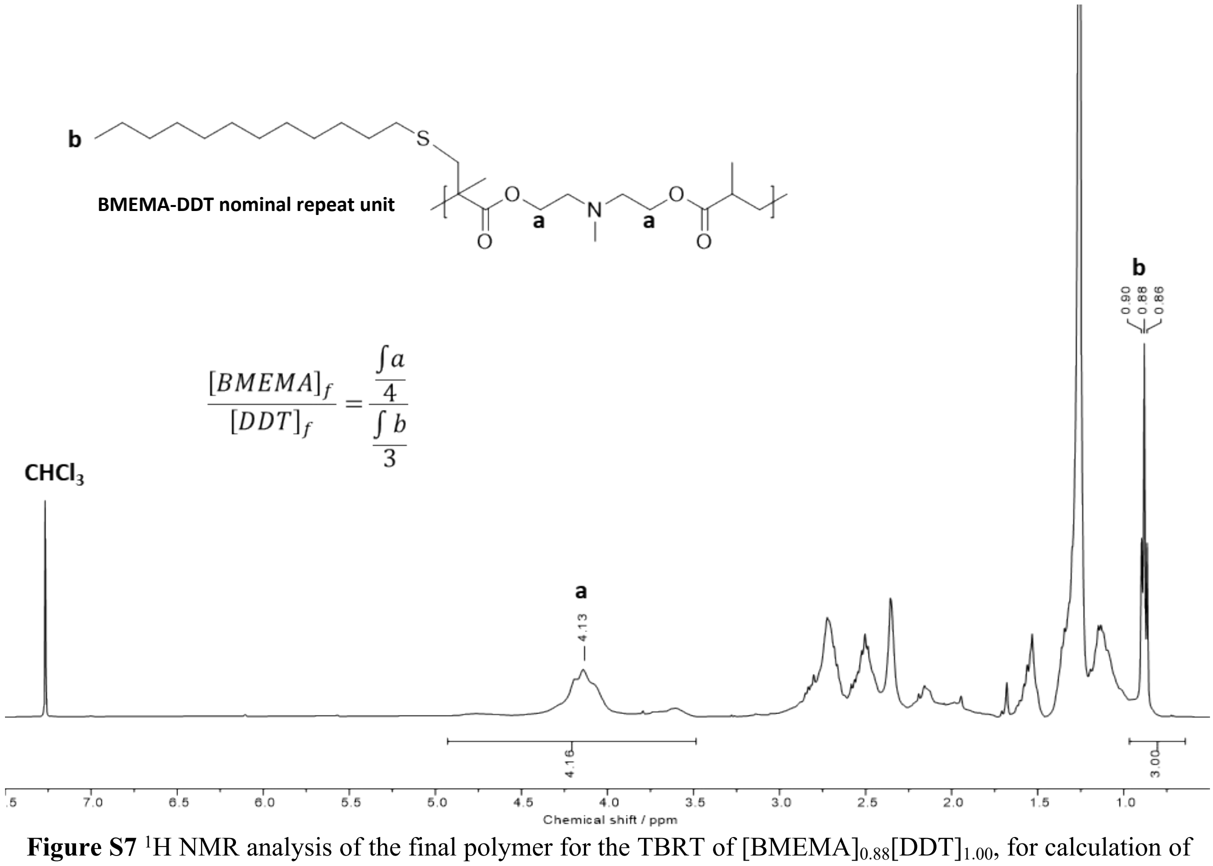

Figure S7 ^{1}H NMR analysis of the final polymer for the TBRT of $[BMEMA]_{0.88}[DDT]_{1.00}$, for calculation of final polymer composition.

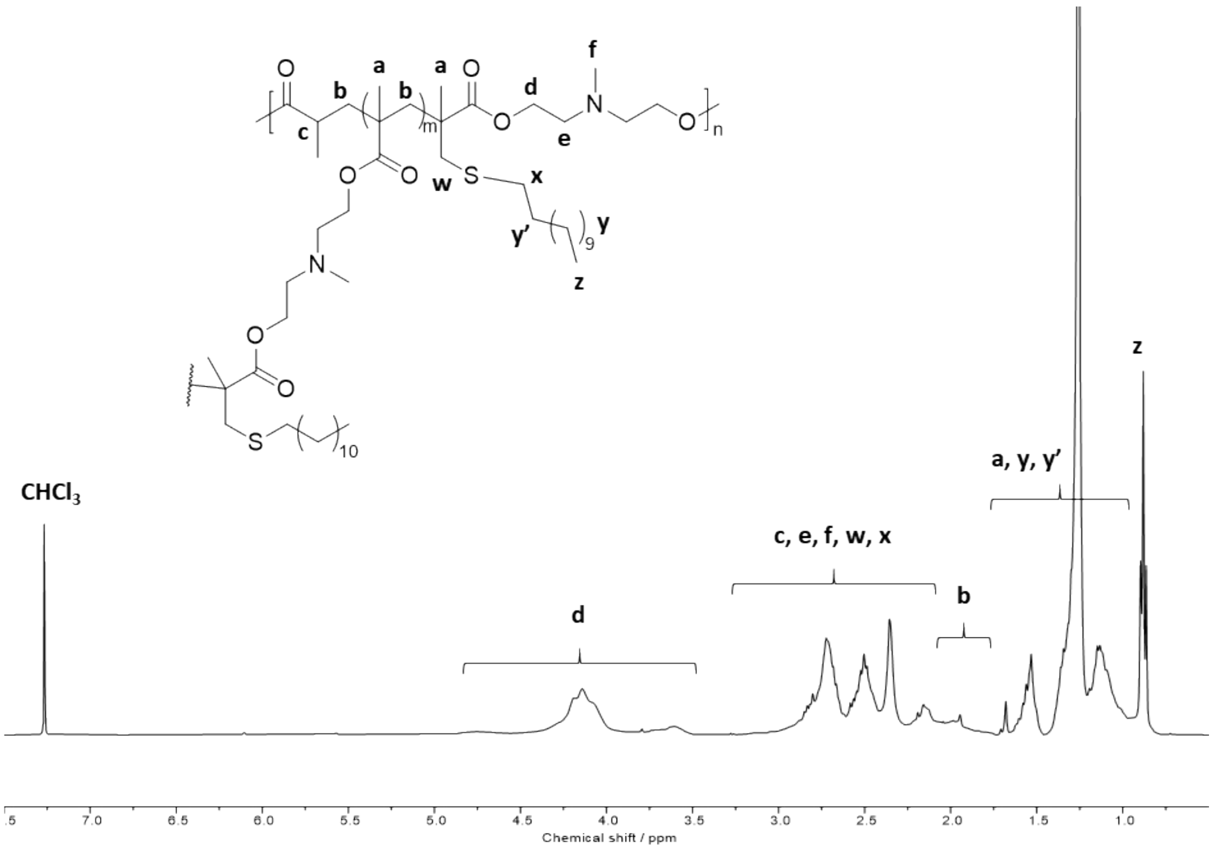

Figure S8 ^{1}H NMR spectral assignments for a branched *p*(DDT-BMEMA) formed by TBRT.

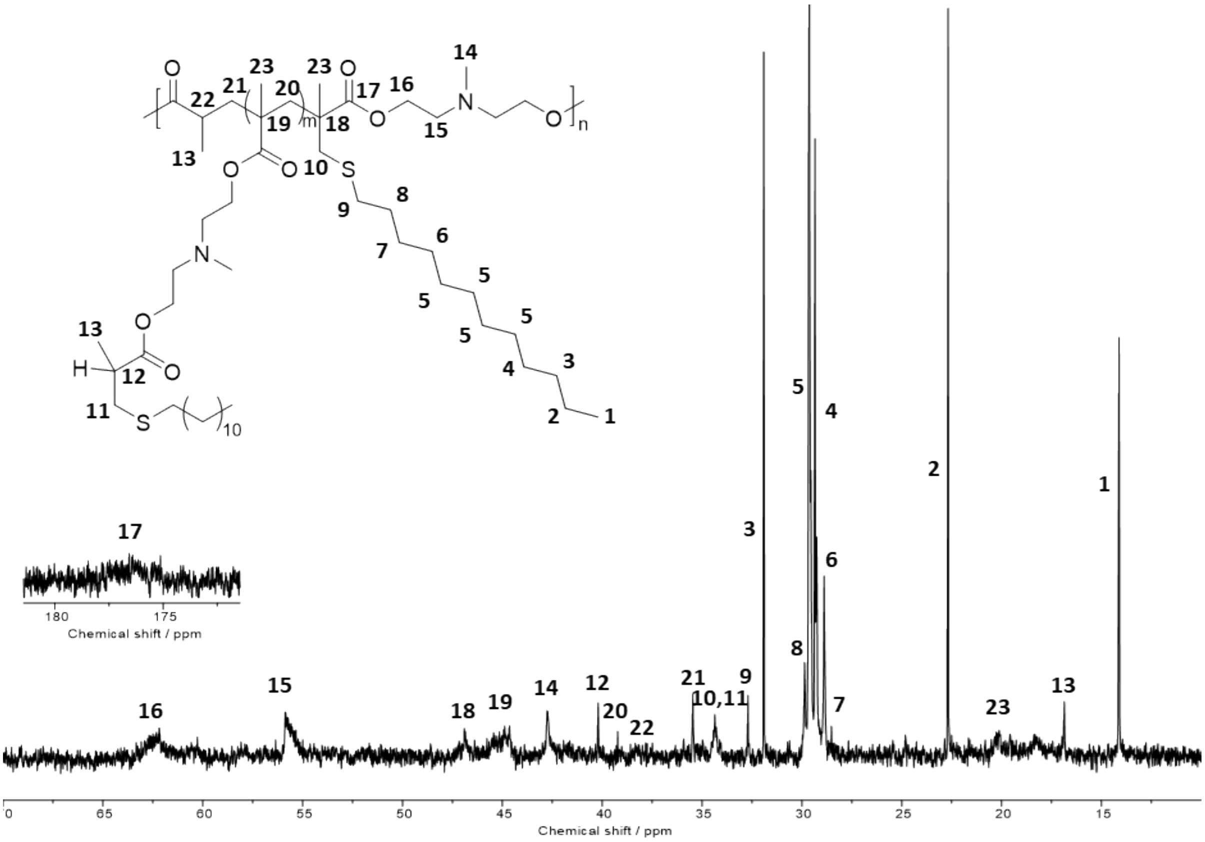

Figure S9 ^{13}C NMR spectral assignments for a branched *p*(DDT-BMEMA) formed by TBRT.

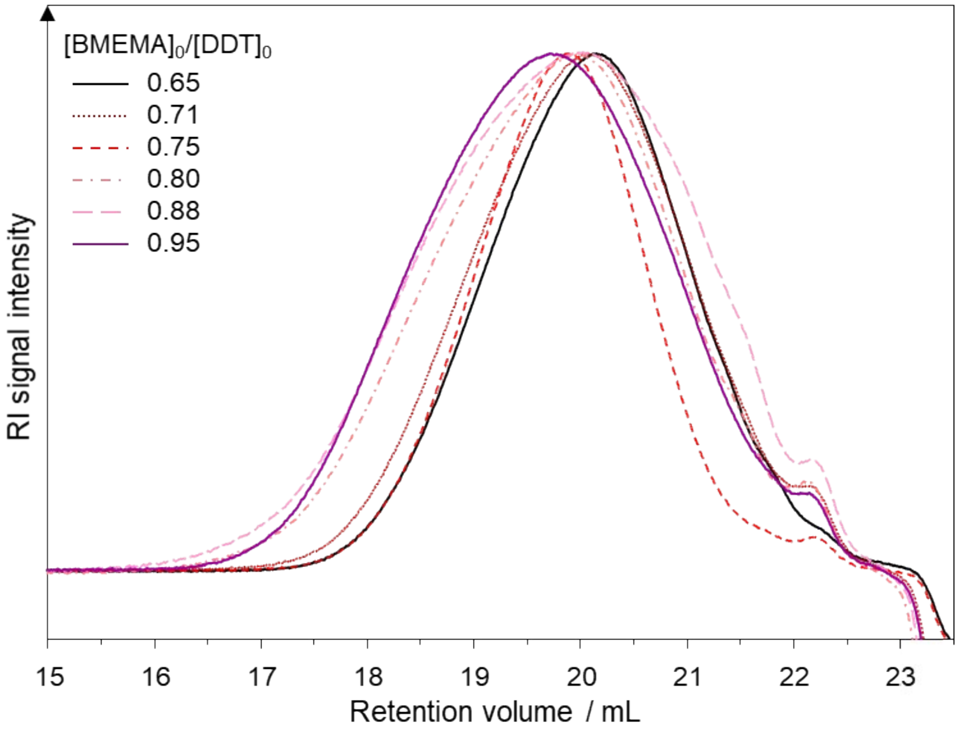

Figure S10 Overlaid refractive index traces for branched *p*(DDT-BMEMA) formed under varying feedstock $[BMEMA]_0/[DDT]_0$ ratios. Peak heights have been normalised to the maximum of each trace.

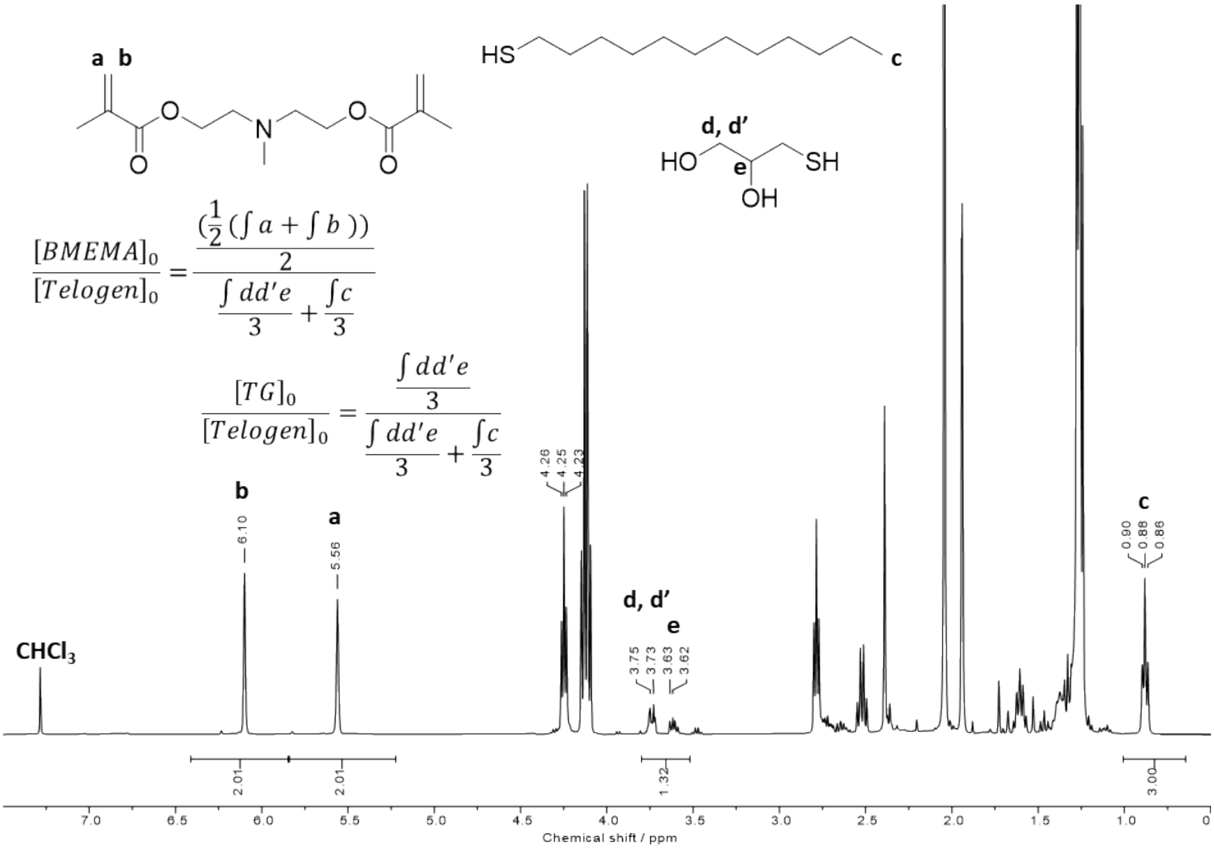

Figure S11 ^{1}H NMR analysis taken at t_0 for a TBRT of BMEMA with TG and DDT, targeting a $[BMEMA]_0/[Telogen]_0$ ratio of 0.70 and a [TG]:[DDT] incorporation of 0.3:0.7 (Table 1).

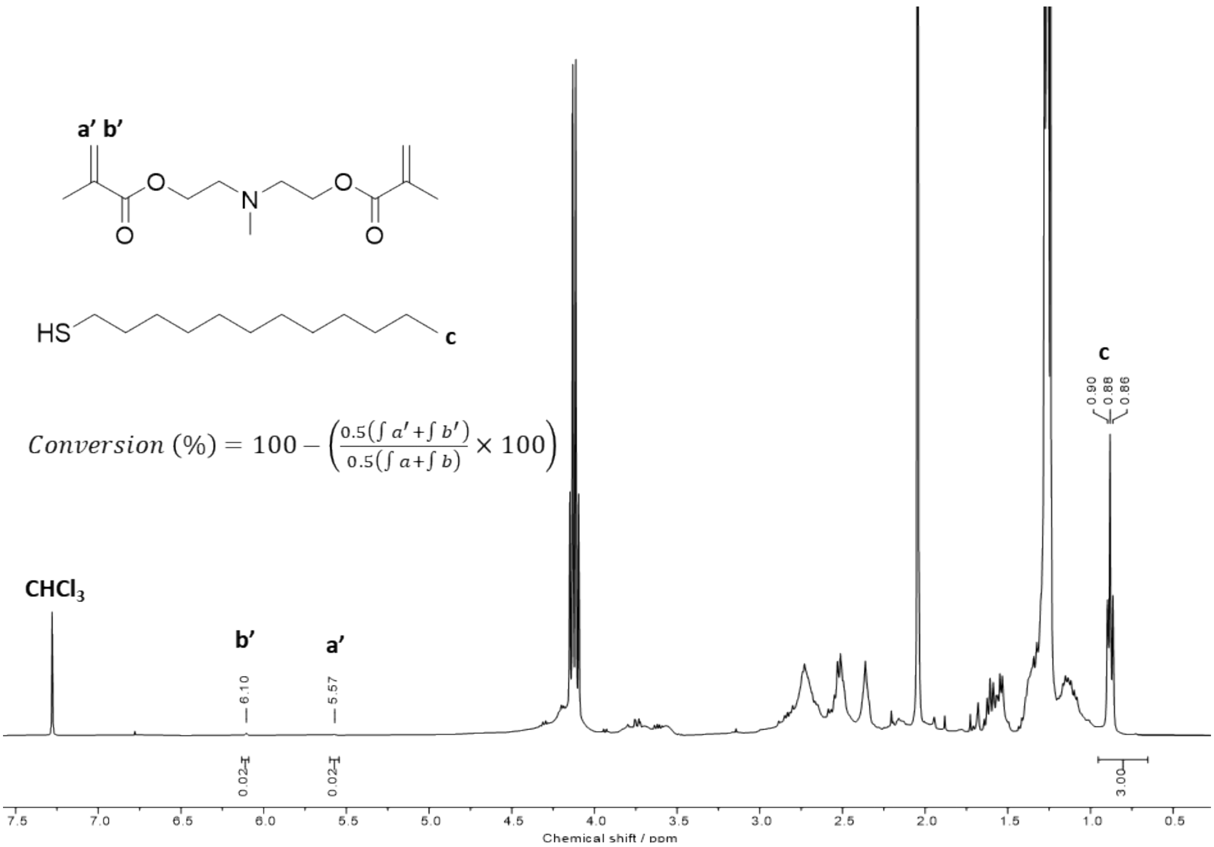

Figure S12 ^{1}H NMR analysis of the crude product for a TBRT of BMEMA with TG and DDT, showing an example calculation for vinyl bond conversion referenced against analysis conducted at t_0 (Figure S11). Signal **c** is utilised as a common integral calibrant.

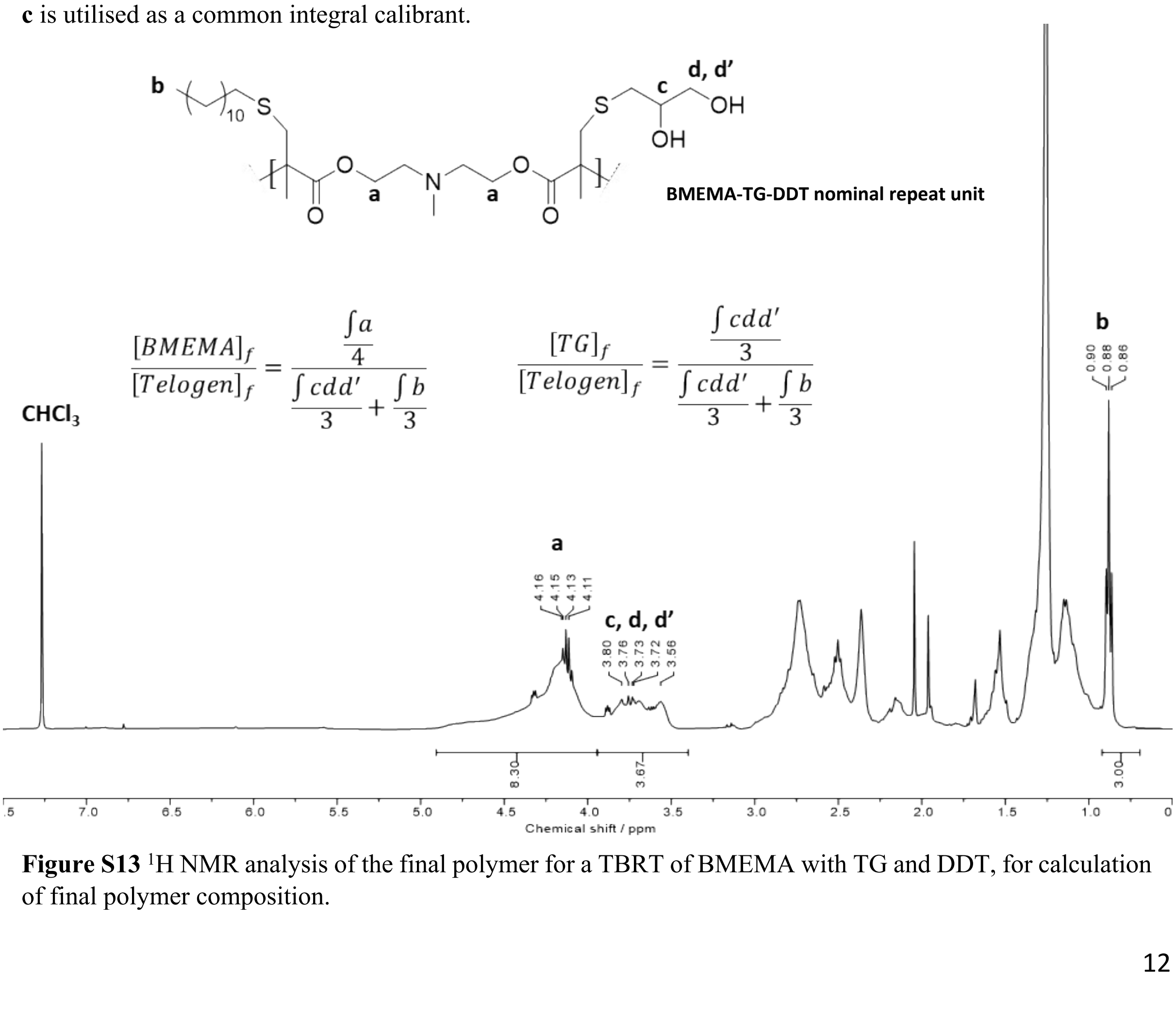

Figure S13 ^{1}H NMR analysis of the final polymer for a TBRT of BMEMA with TG and DDT, for calculation of final polymer composition.

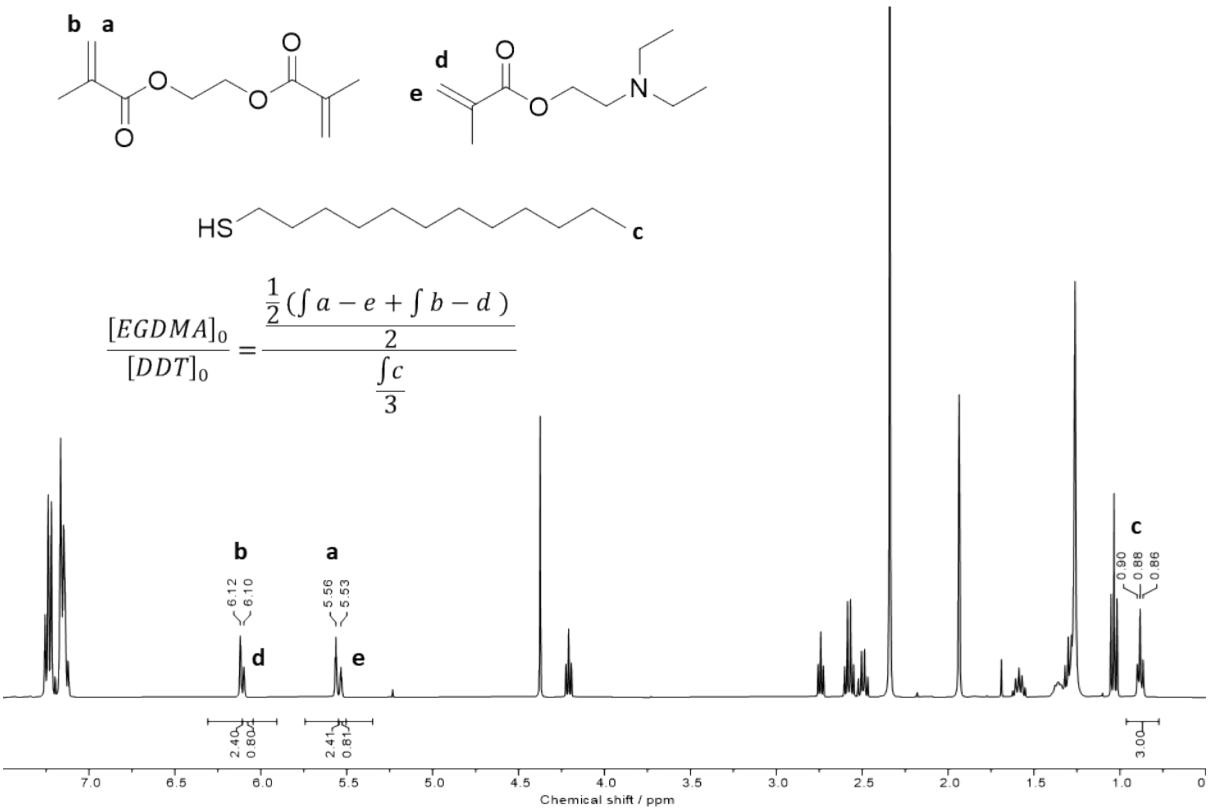

Figure S14 ^{1}H NMR analysis taken at t_0 for a TBRT of EGDMA and DEAEMA with DDT, targeting a $[EGDMA]_0/[DDT]_0$ ratio of 0.80 and an [EGDMA]:[DEAEMA] incorporation of 1:1 (Table 2).

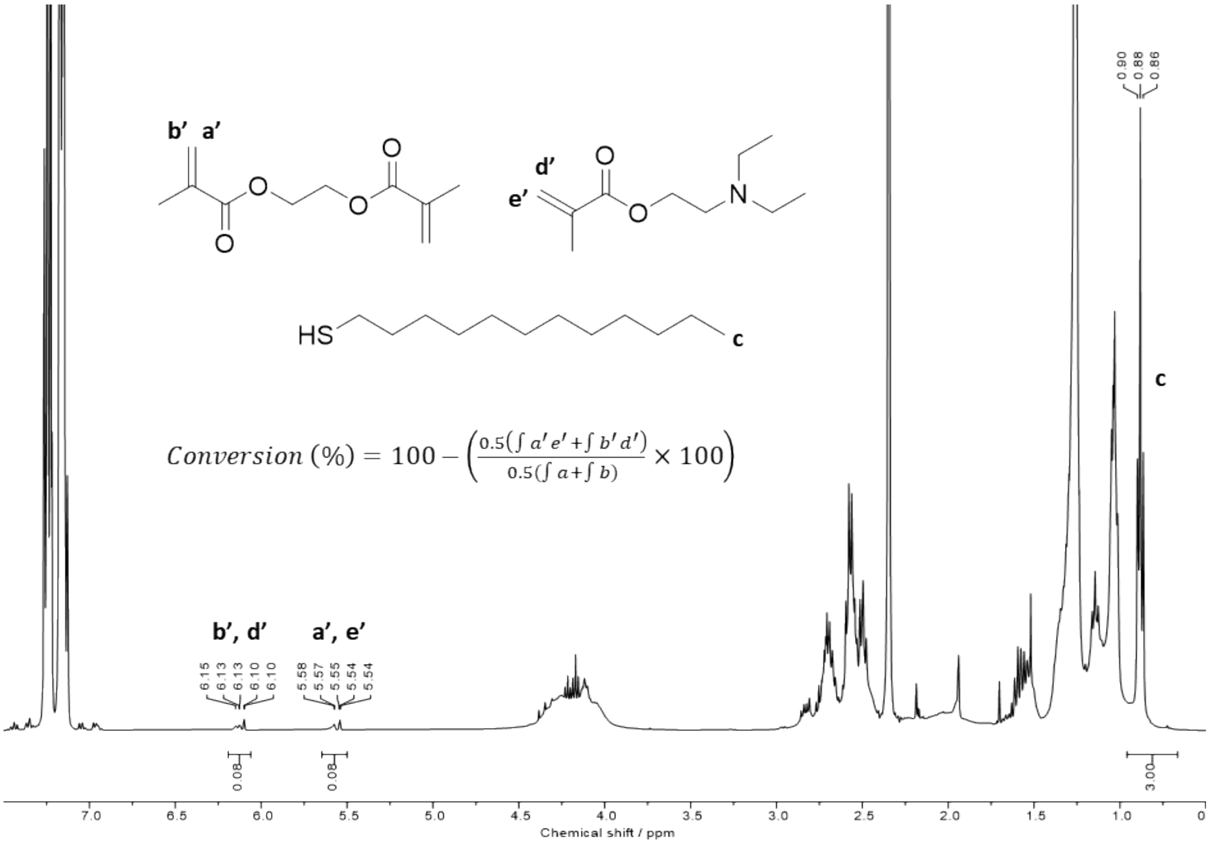

Figure S15 ^{1}H NMR analysis of the crude product for a TBRT of EGDMA and DEAEMA with DDT, showing an example calculation for vinyl bond conversion referenced against analysis conducted at t_0 (Figure S14). Signal **c** is utilised as a common integral calibrant.

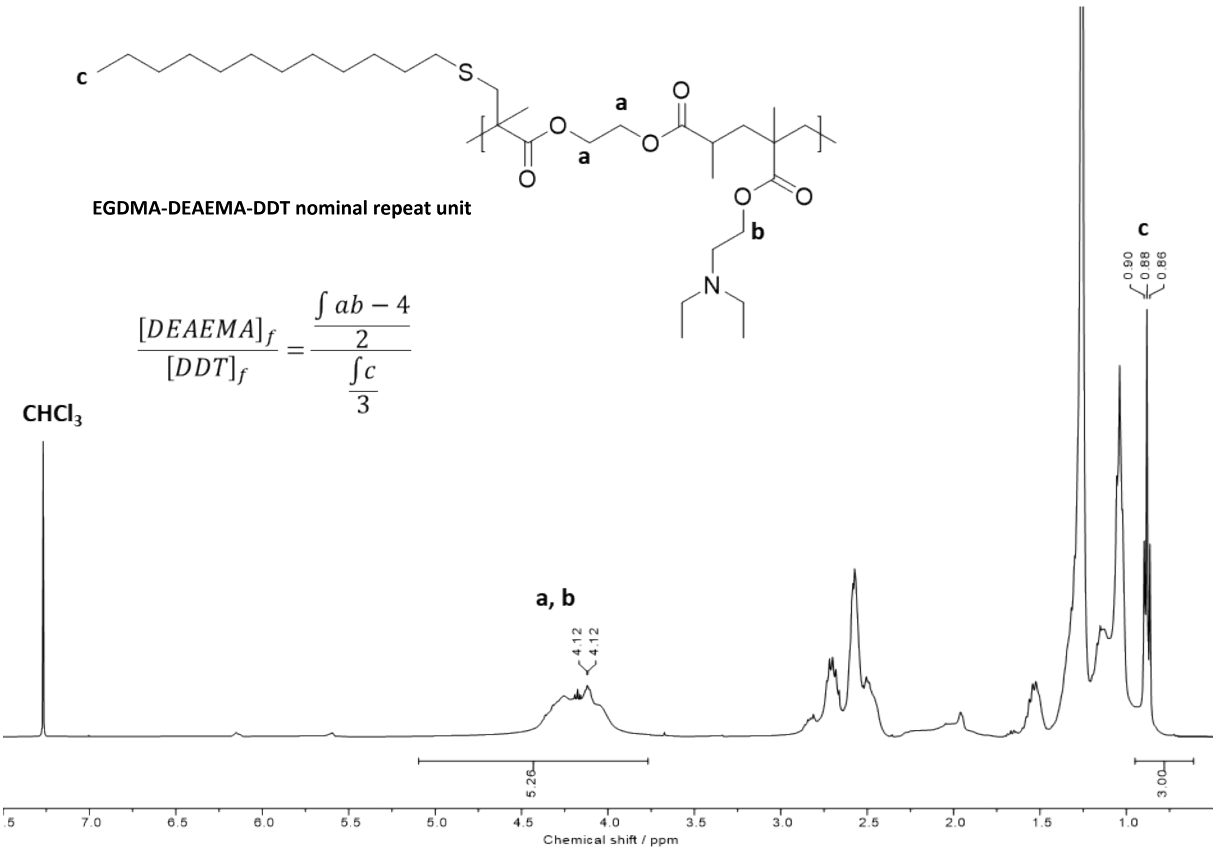

Figure S16 ^{1}H NMR analysis of the final polymer for a TBRT of EGMDA and DEAEMA with DDT, for calculation of final polymer composition. Calculation made based on assumption of 1:1 incorporation of EGDMA:DDT.

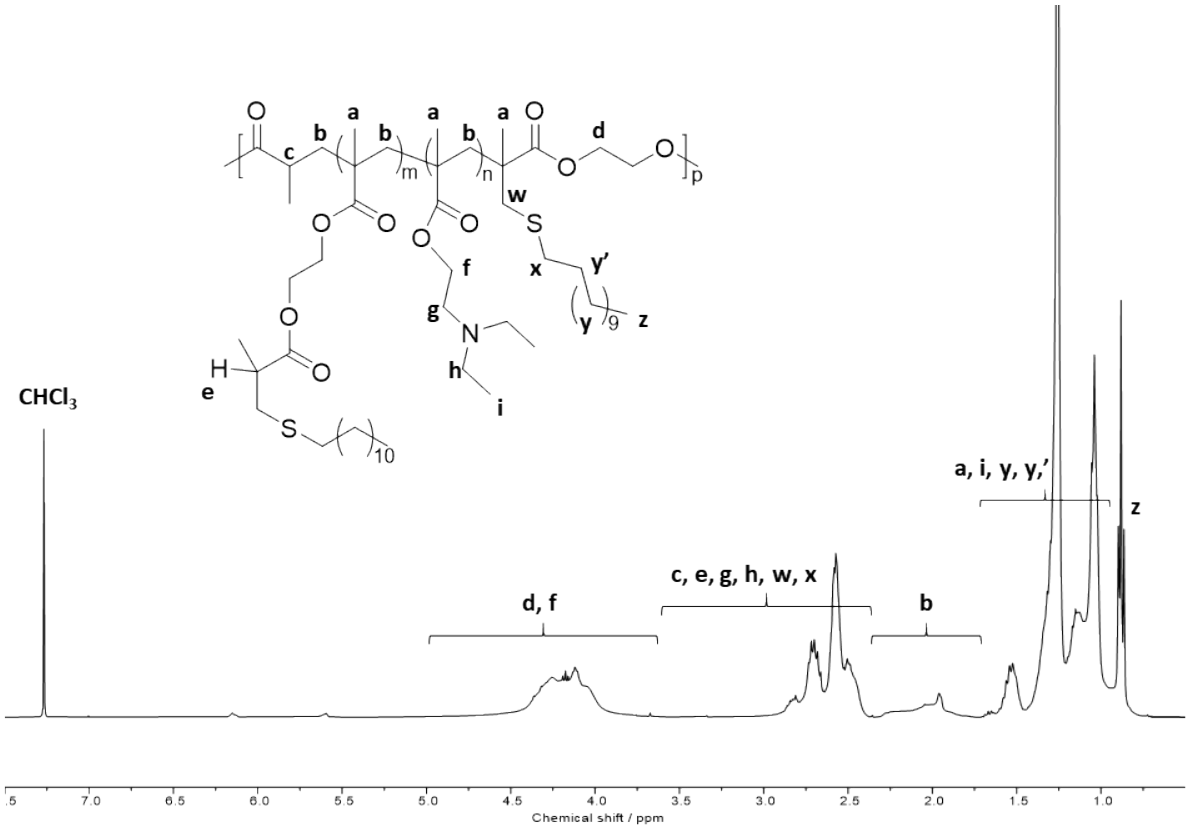

Figure S17 ^{1}H NMR spectral assignments for a branched *p*(DDT-EGDMA-DEAEMA) formed by TBRT.

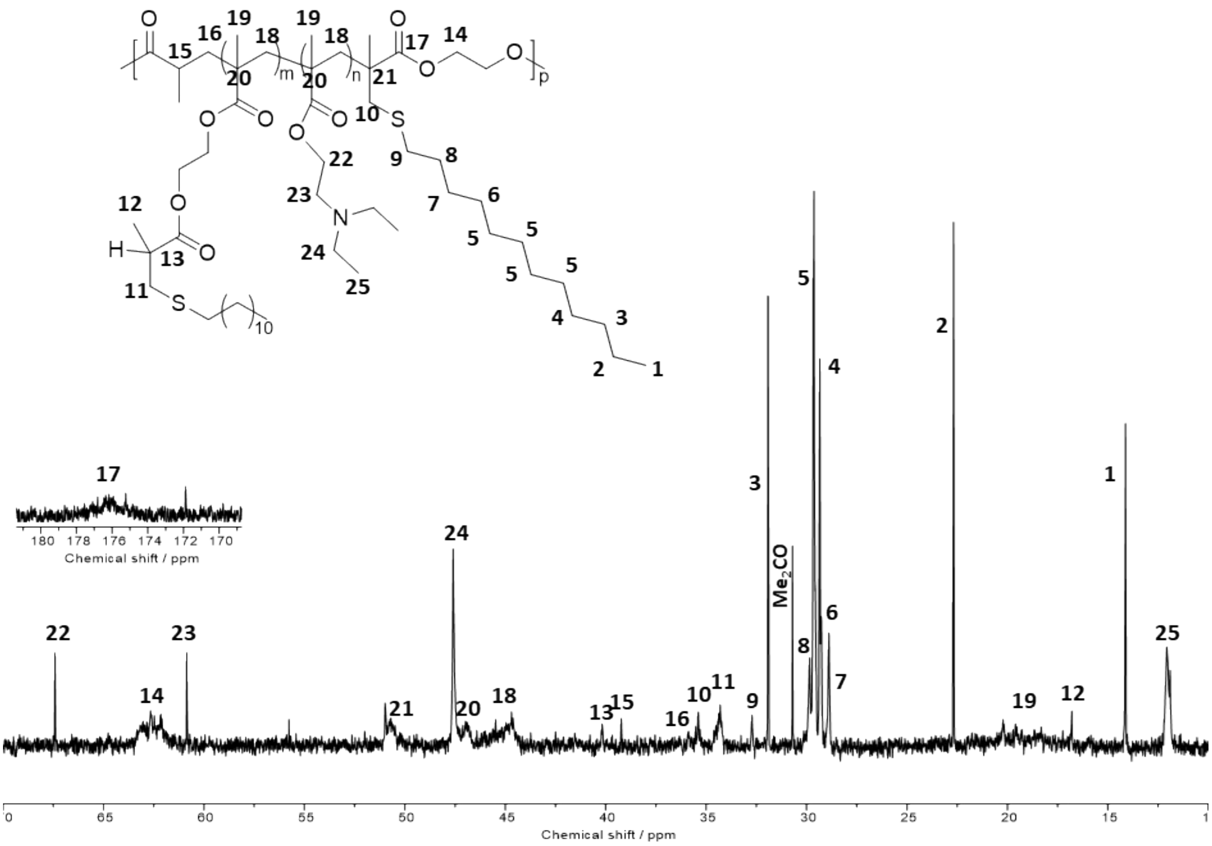

Figure S18 ^{13}C NMR spectral assignments for a branched *p*(DDT-EGDMA-DEAEMA) formed by TBRT.

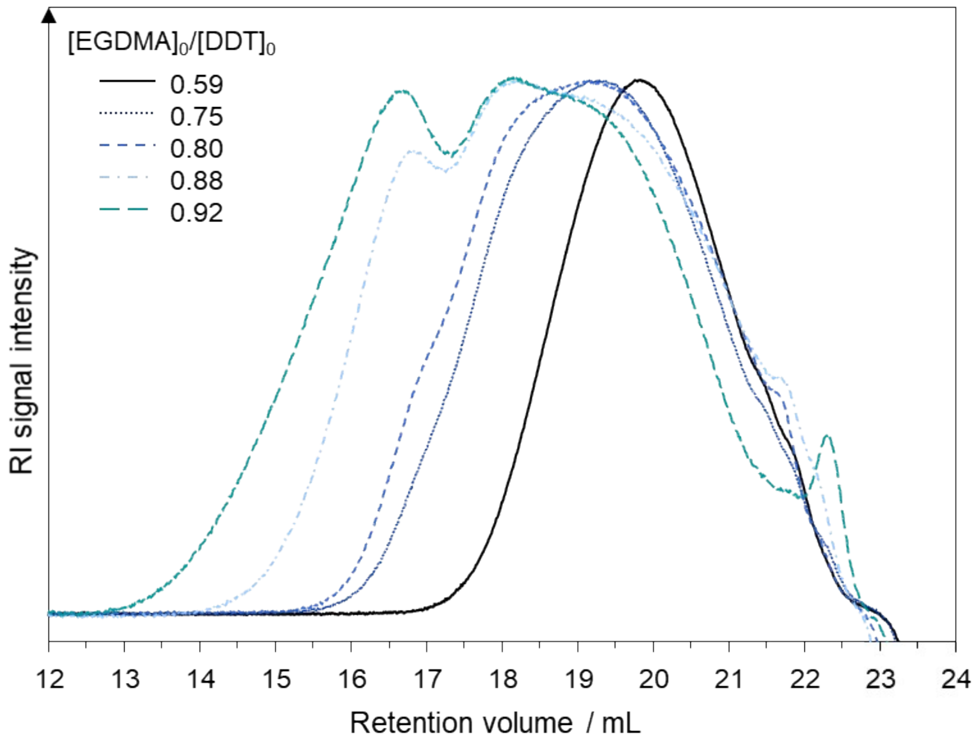

Figure S19 Overlaid refractive index traces for branched *p*(DDT-EGDMA-DEAEMA) formed under varying feedstock $[EGDMA]_0/[DDT]_0$ ratios. Peak heights have been normalised to the maximum of each trace.

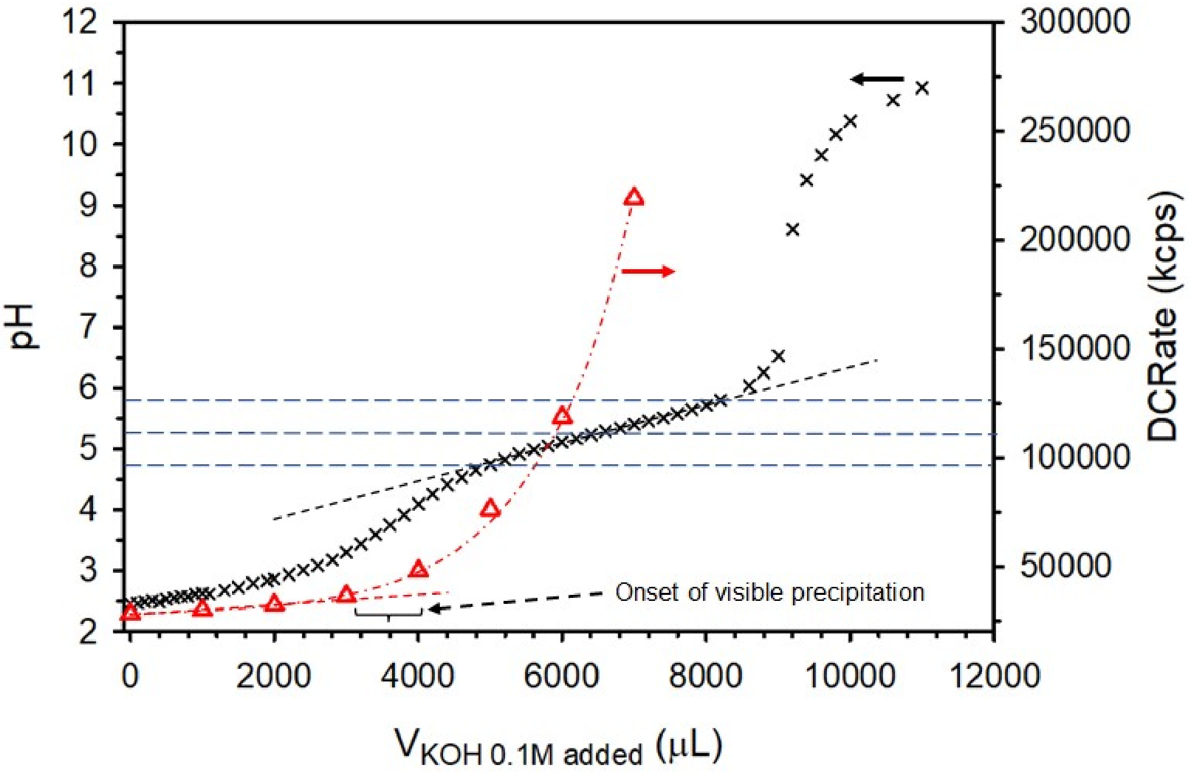

Figure S20 Hydrogen ion titration of p(DDT-EGDMA-DEAEMA) showing variation of pH with added KOH(aq) (black crosses) and impact of KOH addition on derived count rate (red triangles) as measured by dynamic light scattering. A pKa = 5.3 is observed.

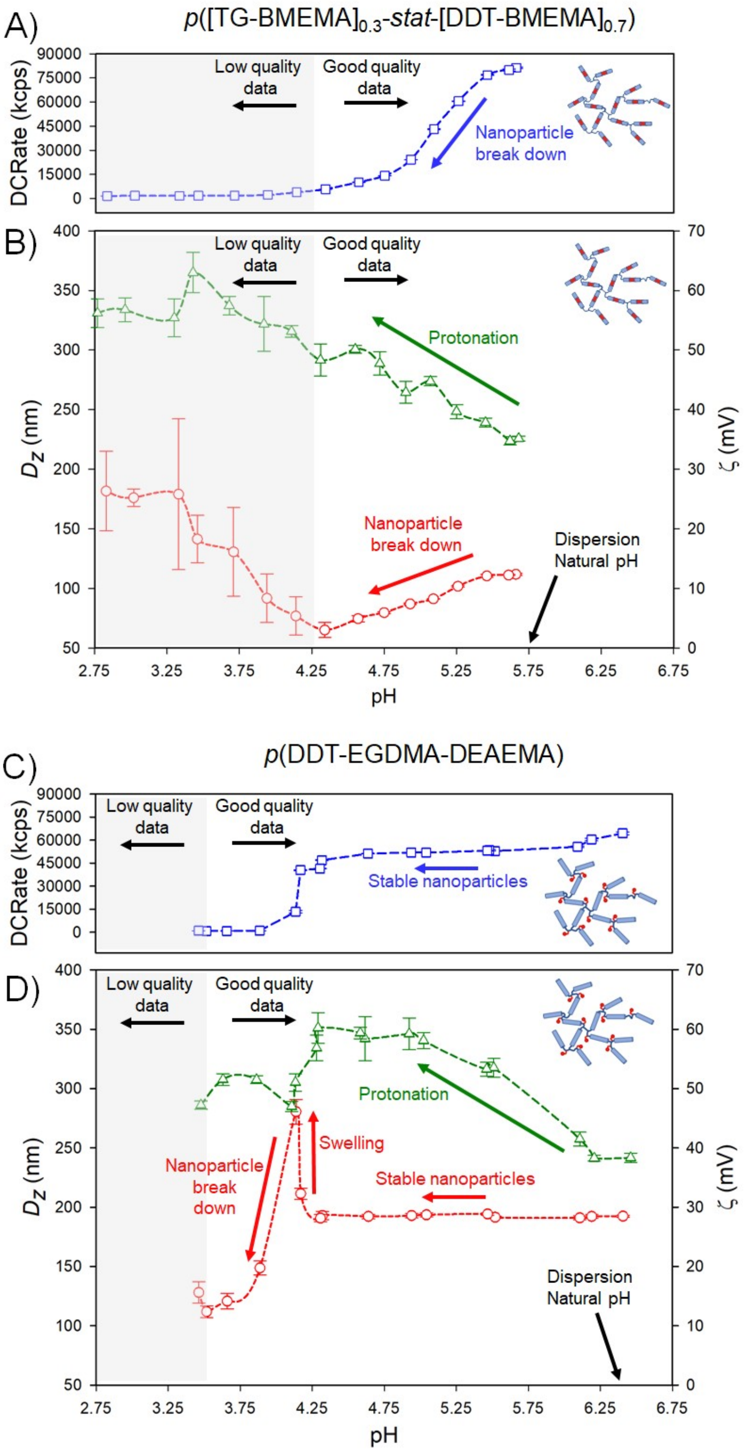

Figure S21 Comparative overlay of dynamic light scattering data from studies of the impact of HCl on aqueous dispersions of TBRT polymer nanoprecipitates. A) Impact of decreasing pH on derived count rate (DCRate) of the nanoparticle dispersion derived from the mixed telogen statistical copolymer p([TG-BMEMA]$_{0.3}$-stat-[DDT-BMEMA]$_{0.7}$); B) impact of decreasing pH on the observed nanoprecipitate z-average diameter (red circles) and zeta potential (green triangles);C) Impact of decreasing pH on derived count rate (DCRate) of the nanoparticle dispersion derived from the mixed mono-vinyl/multi-vinyl taxogen statistical copolymer p(DDT-EGDMA-DEAEMA); D) impact of decreasing pH on the observed nanoprecipitate z-average diameter (red circles) and zeta potential (green triangles); Data are displayed across an identical pH range to allow direct comparison and areas in grey refer to low quality DLS data due to poor light scattering. Apparent increases in size (for example seen in B)) are due to very low scattering rates and anomalies within the liquid media coupled to the r^6 relationship of scattering and measured D_z values.
